# Estimating within-study covariances in multivariate meta-analysis with multiple outcomes

**DOI:** 10.1002/sim.5679

**Published:** 2012-12-03

**Authors:** Yinghui Wei, Julian PT Higgins

**Affiliations:** MRC Biostatistics Unit, Institute of Public HealthRobinson Way, Cambridge CB2 0SR, U.K.

**Keywords:** multivariate meta-analysis, correlated outcomes, nested events, delta method, within-study correlation

## Abstract

Multivariate meta-analysis allows the joint synthesis of effect estimates based on multiple outcomes from multiple studies, accounting for the potential correlations among them. However, standard methods for multivariate meta-analysis for multiple outcomes are restricted to problems where the within-study correlation is known or where individual participant data are available. This paper proposes an approach to approximating the within-study covariances based on information about likely correlations between underlying outcomes. We developed methods for both continuous and dichotomous data and for combinations of the two types. An application to a meta-analysis of treatments for stroke illustrates the use of the approximated covariance in multivariate meta-analysis with correlated outcomes. Copyright © 2012 John Wiley & Sons, Ltd.

## 1. Introduction

Meta-analysis combines the results from multiple studies that addresses similar research questions, and is frequently applied to randomized trials to investigate the effects of specific interventions. In many cases, studies report results for more than one outcome, or report repeated measures on a particular outcome 1, and it is not uncommon for these outcomes to be correlated. For example,

- Correlation may arise from correlated outcomes measured on the same participants, such as math and reading scores in school examinations 2, or probing depth and attachment level in the treatment of periodontal disease 3. Later in the paper, we discuss an example from a meta-analysis of the effects of vasoactive drugs on acute stroke 4, involving the correlated outcomes of systolic and diastolic blood pressures.
- Correlation may arise when one event is nested within another 4,5. The example later in the paper reports outcomes of ‘death’ and ‘death or disability’, the former being nested within the latter.
- Correlation may arise from outcomes measured repeatedly on the same participants, such as survival rates at 3-year follow-up and at 5-year follow-up after treatment for breast cancer 6.
- Correlation may arise when one outcome is a surrogate marker for another, such as CD4+ cell count as a surrogate marker for AIDS 7.

The effects of interventions are measured using treatment effect estimates, such as standardized differences in means, or odds ratios, applied separately for each outcome. When the outcomes are correlated, the treatment effect estimates are also correlated within a study. These dependencies between treatment effect estimates are ignored if multiple univariate meta-analyses are performed, as is typically done in practice.

A multivariate meta-analysis approach may be used to incorporate these nonzero within-study correlations, thereby improving the precision of the analysis compared with using a univariate approach. Kirkham *et al.* 8, Riley *et al.* 9, and Riley 10 have demonstrated such improvements in precision, although in practice the improvements can be small. In contrast, Kirkham *et al.* 8 have demonstrated a notable advantage of a multivariate approach when there is selective nonreporting of outcomes on the basis of the statistical significance of the treatment effect estimates. Specifically, by exploiting the correlation between reported and nonreported outcomes, one can substantially reduce biases due to selective reporting. Such outcome reporting bias is now well documented in the medical literature 11, 12, so multivariate approaches to meta-analysis have a promising future. The multivariate approach also allows correlations in treatment effects across studies to be estimated as part of a random-effects model. This facilitates the computation of joint prediction regions for treatment effects in new studies, as is used in bivariate meta-analysis of diagnostic accuracy studies 13.

A standard multivariate meta-analysis approach assumes that the within-study variances and covariances for the treatment effect estimates are known. A well-recognized problem in the implementation of multivariate methods is how to estimate the within-study covariances, because they are rarely available in published study reports 10,14–16. Our objective in this paper is to develop an approach to estimation of within-study covariances between treatment effect estimates. In particular, we introduce, derive, and apply a series of asymptotic estimators of within-study covariance for different treatment effect measures. In Section 2, we briefly review the multivariate meta-analysis model as it applies to correlated outcomes. In Section 3, we derive approximate formulae for within-study covariance. In Section 10, we discuss practical issues, and in Section 13, we perform a simulation study to investigate the properties of our methods. In Section 16, we apply our methods to data from a systematic review of trials of vasoactive drugs for acute stroke. We close with discussion in Section 20.

## 2. Multivariate random-effects meta-analysis

The conventional random-effects multivariate meta-analysis model based on treatment effect estimates has a hierarchical structure with multivariate normal distributions at each of two levels, corresponding to within-study and between-study components. We let *m* denote the number of outcomes of interest and use 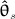 to denote a vector of observed treatment effect estimates from study *s*. Because several outcome variables are measured on the same individual within a study, the entries in 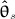 may be correlated and we assume the vector follows a multivariate normal distribution:


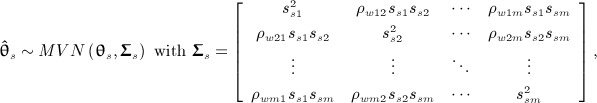


where ***θ***_*s*_ is the vector of underlying true treatment effects for the outcomes within study *s*. We assume these to be normally distributed and centered around ***θ*** = (*θ*_1_,*θ*_2_, …, *θ*_*m*_)^*T*^, the true average treatment effects across studies for the set of outcomes:


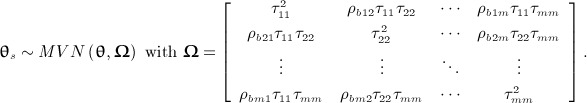


The matrix **Σ**_*s*_ refers to within-study covariance. Its diagonal entries are the sampling variances 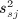 for the treatment effect estimates for each outcome *j*, and off-diagonal entries represent the covariance between treatment effect estimates for pairs of outcomes within the study, reflecting the correlation that arises when several outcomes are measured on the same participants within the study. The matrix ***Ω*** represents the between-study covariance. Heterogeneity variances 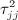 (*j* = 1, …, *m*) for true treatment effects comprise the diagonal elements. The off-diagonal elements reflect the correlation arising when the same outcomes are also measured by other studies. When the within-study correlations *ρ*_*w*.._ and between-study correlations *ρ*_*b*.._ are all zero, the model is equivalent to several separate univariate random-effects models.

The objective of a multivariate random-effects meta-analysis is to estimate the mean treatment effects across studies, ***θ*** = (*θ*_1_,*θ*_2_, …, *θ*_*m*_), and the between-study covariance matrix, ***Ω***. The full specification of the multivariate model requires knowledge of the within-study correlations, *ρ*_*w*.._. Given values for these correlations, the estimation methods for the model are straightforward using, for example, SAS Proc Mixed 17, the Stata program *mvmeta* 18 with estimation options including (restricted) maximum likelihood estimate and method of moments 19, or a Bayesian approach in WinBUGS 20.

## 3. Estimating within-study covariance

The methods described in Section 2 assume that the within-study covariance matrix is known. If the within-study correlation coefficients between treatment effects are not known, they might be borrowed from studies that provide individual participant data; examples include application to rheumatoid arthritis data 21 and periodontal disease data 17,21,22. When individual participant data are not available, multivariate meta-analysis can be carried out by assuming a plausible value for each unknown correlation coefficient 16,17. These plausible values might be derived using clinical considerations. Alternatively, a recent proposal is to use the empirical correlation coefficient observed between treatment effect estimates across studies 8. Furthermore, a Bayesian framework can be used in which prior distributions are placed on the correlation coefficients; Nam *et al*. 23 used a noninformative uniform, U[-1,1], distribution 10.

Most imputation approaches have been based on imputing the correlation between treatment effect estimates and assuming this correlation to be identical for every study. For example, if each study contributes odds ratio estimates for two correlated outcomes, a correlation coefficient between the estimated treatment effects (as log odds ratios) would be imputed and the same value would be used for each study. In contrast, in this paper, we focus on the correlation between the outcomes themselves. The distinction is that the outcome is the direct measurement on the participants, whereas the treatment effect is a quantity that describes the benefit or harm of the treatment when compared with control. There are two potential advantages to evaluating within-study correlation at the outcome level. First, these correlations are more likely to be known from external sources than correlations between treatment effects, and if not, then plausible values for them are more readily provided than between treatment effect estimates. Second, these correlations are more natural descriptors of inherent similarities and allow the correlations between treatment effect estimates to vary according to other measurable features of the study.

We consider both continuous and dichotomous outcomes, which are the most common in meta-analysis. We begin with the covariance between two estimates of mean differences, because this can be derived in closed form. For the other covariances, we implement a bivariate delta method. From here forward, we drop the subscript *s* for study.

### 3.1. Covariance between two mean differences

Consider a study with *N*_*t*_ and *N*_*c*_ participants in treatment and control groups, respectively. Each group reports measures on both outcome 1 and outcome 2. Note that not all participants need necessarily contribute data on both outcomes. We use *n*_1*t*_, *n*_2*t*_, and *n*_12*t*_ to denote the number of participants who report outcome 1, of those who report outcome 2, and of those who report both outcome 1 and outcome 2, respectively, in the treatment group. These numbers are such that *n*_12*t*_ *≤ n*_1*t*_ *≤ N*_*t*_ and *n*_12*t*_ *≤ n*_2*t*_ *≤ N*_*t*_. In a similar way, we define *n*_1*c*_, *n*_2*c*_, and *n*_12*c*_ for the control group.

We assume that the outcome variable *y*_*jai*_ for participant *i*, outcome *j*, and arm *a* is marginally normally distributed:


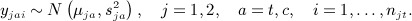


Given a known correlation coefficient *ρ* between the two outcomes themselves, and assuming it is the same in both treatment groups, the following give the analytical form for the covariance between mean differences (*MD*_1_,*MD*_2_):


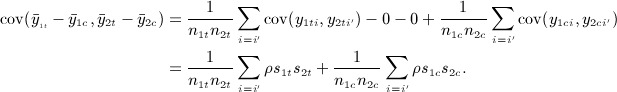
(1)

Therefore,


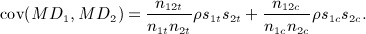
(2)

We describe some simplifying assumptions in Section 4.1.

### 3.2. Bivariate delta method

Estimation of covariances other than between two mean differences requires the use of the delta method 24, because an analytic solution is not possible. A bivariate delta method considers two functions of a common set of random variables, and we can use this to compute asymptotic covariance matrix of the two functions by first-order Taylor expansion approximation.

Consider a vector **X** = (*X*_1_,*X*_2_, …, *X*_*p*_)^*T*^ of *p* random variables, with mean ***μ*** = (*μ*_1_,*μ*_2_, …, *μ*_*p*_) and *p* × *p* variance–covariance matrix Σ*. Let *f*_1_(**X**) and *f*_2_(**X**) denote functions of **X**. We can approximate the variance of **Y** = (*f*_1_(**X**),*f*_2_(**X**)) ^*T*^ through first-order Taylor expansion,


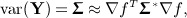
(3)

where ∇ *f* is the *p* × 2 matrix of partial derivatives of functions *f*_*i*_ with respect to *X*_*j*_ evaluated at the point *E*(*X*_*i*_),


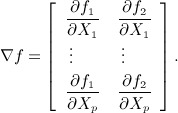
(4)

To implement these ideas, we identify **Y** with the pair of treatment effect estimates 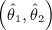 for which the covariance is required. Computation of the asymptotic covariance matrix between these two treatment effect estimates, expressed as the functions of basic statistics *f*_1_(**X**) and *f*_2_(**X**), requires specification of functions *f*_1_ and *f*_2_ and the distribution of random variables **X**, which need to be either estimated or known asymptotically. The approximation error of the bivariate delta method is the remainder from the first-order bivariate Taylor expansion. The remainder is a function of **X**^2^ and the second derivative of the two functions *f*_1_(**X**) and *f*_2_(**X**) with respect to **X**. The analytical expression has a complex form and can be difficult to derive in practice as it depends on the specification of *f*_1_(**X**), *f*_2_(**X**), and **X**. Instead, we assess the approximation error using Monte Carlo simulation in Section 16.

### 3.3. Covariance between two log odds ratios

Treatment effects for dichotomous outcomes in meta-analysis are usually measured using odds ratios, risk ratios or risk differences. If two dichotomous outcomes are considered, occurrences of events are likely to be associated, so the covariance between the two treatment effect measures will be nonzero. In this section, we consider the odds ratio, which provides a relatively straightforward application of the bivariate delta method to the derivation of a covariance. We first consider the general case of two different dichotomous outcomes and then consider the special case that one outcome is nested within the other. Trikalinos and Olkin 25 have derived an approximate covariance matrix for two dichotomous outcomes that are mutually exclusive of each other.

We write a pair of log odds ratios as functions of the random vector 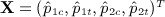. In the notation of Section 3.2, we take


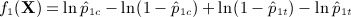


and


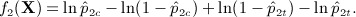


We then use the bivariate delta method to obtain the covariance matrix for **Y** = (*f*_1_(**X**),*f*_2_(**X**)) ^*T*^ and hence the desired covariance between the two log odds ratios.

The expectation of **X** is simply the vector of true risks, (*p*_1*c*_,*p*_1*t*_,*p*_2*c*_,*p*_2*t*_)^*T*^. The covariance of **X** is given by


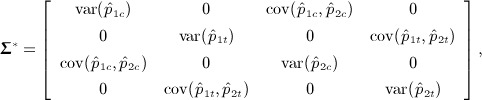


where we assume that outcomes from different treatment arms are measured on different participants and are therefore independent, so that 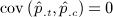. The diagonal entries are given by


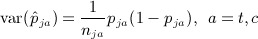
(5)

for outcomes *j* = 1 or 2. We show in Section A1 of our supplementary materials ‡ that the covariance term is


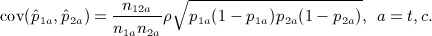
(6)

Implementing the bivariate delta method (Equation (3)), we obtain


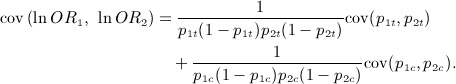
(7)

In practice, the probabilities (risks) would be substituted by their maximum likelihood estimates 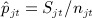 and 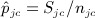, where *S*_*jt*_ and *S*_*jc*_ are the numbers of participants with the outcome *j* event in the treatment and control groups, respectively. By substituting (6), and writing *F*_*jt*_ and *F*_*jc*_ for the respective numbers without the event, we can write the covariance (7) as


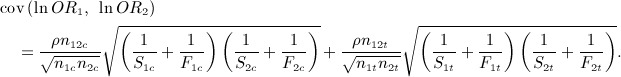
(8)

We can derive an alternative to Equation (8) for the case when one outcome is nested within the other, for example, when jointly analyzing ‘death’ and ‘death or disability’. In Section A2 of our supplementary materials, we show that if outcome 1 is nested within outcome 2, the covariance term for this special case is


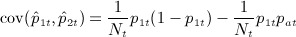
(9)

with *p*_*at*_ being the probability of *additional* events. Similarly, we derive the formulae for the control group. The following then provides the desired covariance:


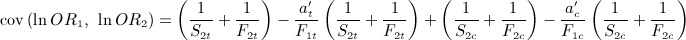
(10)

where *a* ′ _*t*_ = *a*_*t*_ + (*n*_1*t*_ − *n*_2*t*_) × (*S*_2*t*_ − *S*_1*t*_) / (*n*_2*t*_ − *S*_1*t*_) and *a* ′ _*c*_ = *a*_*c*_ + (*n*_1*c*_ − *n*_2*c*_) × (*S*_2*c*_ − *S*_1*c*_) / (*n*_2*c*_ − *S*_1*c*_) with *a*_*t*_ and *a*_*c*_ denoting observed additional numbers of events for outcome 1 rather than 2 in treatment and control groups, respectively. Note that formula (10) is a function of occurrences of events in the two treatment groups and is free of imputation of between-outcome correlation coefficients.

### 3.4. Covariance between two standardized mean differences

Returning to the case of continuous outcome measures, a common metric used in meta-analysis is the standardized mean difference (SMD). Here, we provide the derivation of the covariances between two estimates of Hedges’ *g* 26, which is an unbiased estimate of the SMD. Hedges’ *g* is computed as the mean difference divided by the pooled standard deviation, multiplied by a small-sample bias correction factor:


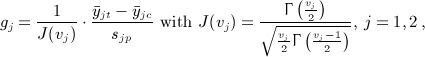


where *s*_*jp*_ is the usual a pooled standard deviation for outcome *j*, given by


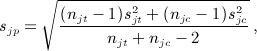


and *v*_*j*_ = *n*_*jt*_ + *n*_*jc*_ − 2. The constant small-sample correction factor, *J*(*v*_*j*_) can be approximated by *J*(*v*_*j*_) = 1 − 3 /(4*v*_*j*_ − 1) 27.

Formulae for the covariance between two estimates of Hedges’ are available for the situation in which complete data are observed for both outcomes 27–30. We derive the covariance between two SMDs for the more general case that allows different numbers of participants to contribute to different outcomes. Setting 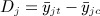, we write **X** = (*D*_1_,1 /*s*_1*p*_,*D*_2_,1 /*s*_2*p*_), so that the two SMDs can be written as functions of **X**: *f*_1_(**X**) = *X*_1_*X*_2_ / *J*(*ν*_1_) and 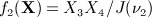.

We then use the bivariate delta method to obtain the covariance matrix for **Y** = (*f*_1_(**X**),*f*_2_(**X**)) ^*T*^ and hence the desired covariance between the two SMDs. The expectation of **X** is given by *E*(**X**) = (*μ*_1*t*_ − *μ*_1*c*_,1 / *J*(*v*_1_)*σ*_1_,*μ*_2*t*_ − *μ*_2*c*_,1 / *J*(*v*_2_)*σ*_2_) ^*T*^, because *E* (1 / *s*_*jp*_) = 1 / *J*(*v*_*j*_)*σ*_*j*_, as White and Thomas derived 28. In the covariance matrix for **X**, the diagonal entries are given by the variances of mean differences and variances of inverses of the sample variances:


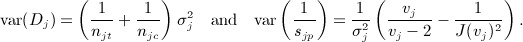


White and Thomas 28 again derived the latter. We assume that mean differences are uncorrelated with variances, so that some off-diagonal entries are zero. The nonzero entries are then


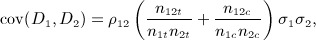
(11)

according to (2), and


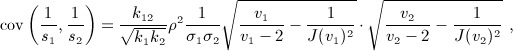
(12)

as demonstrated in Section A3 of our supplementary materials, where *k*_12_, *k*_1_, and *k*_2_ are functions of the sample sizes as defined in Table I. Application of Equation (3) of the bivariate delta method gives the covariance between the two SMDs as


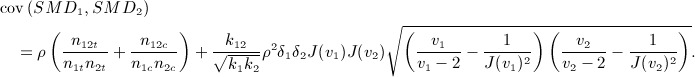


**Table I:** Asymptotic estimators for covariance between various pairs of treatment effect estimates

Effect size for outcome 1	Effect size for outcome 2	Covariance formula	Equation
MD	MD	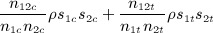	(1.1)
	SMD	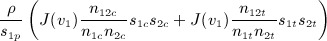	(1.2)
	logOR	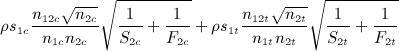	(1.3)
	logRR	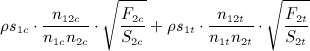	(1.4)
	RD	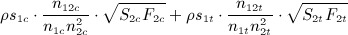	(1.5)
SMD	SMD	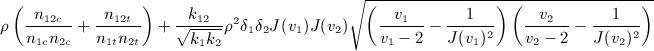	(1.6)
	logOR	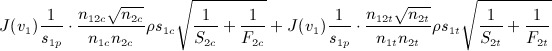	(1.7)
	logRR	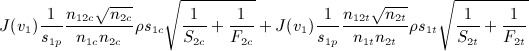	(1.8)
	RD	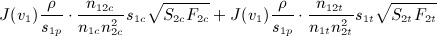	(1.9)
logOR	logOR	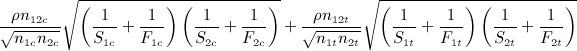	(1.10)
	logRR	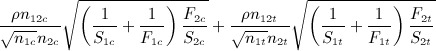	(1.11)
	RD	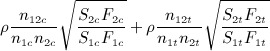	(1.12)
logRR	logRR	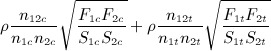	(1.13)
	RD	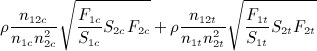	(1.14)
RD	RD	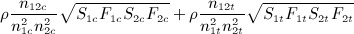	(1.15)

MD: mean differences; SMD: standardized MD; logOR: log odds ratio; logRR: log risk ratio; RD: risk difference; *n*_1*t*_: number of participants reporting outcome 1 in treatment group; *n*_2*t*_: number of participants reporting outcome 2 in treatment group; *n*_12*t*_: number of participants reporting both outcome 1 and outcome 2 in treatment group; *n*_1*c*_, *n*_2*c*_, and *n*_12*c*_: defined in the similar way for control group; 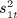: sample variance for outcome 1 in treatment group; 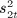: sample variance for outcome 2 in treatment group; 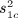 and 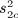: defined in a similar way for control group; *s*_*jp*_: pooled standard deviation for outcome *j*; *J*(*v*): a small-sample correction factor for SMD, *J*(*v*) = 1 − 3 / (4*v* − 1); 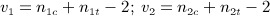; *S*_1*t*_: number of participants with event for outcome 1 (dichotomous) in treatment group; *F*_1*t*_: number of participants without event for outcome1 (dichotomous) in treatment group; *S*_1*c*_ and *F*_1*c*_: defined in a similar way for control group; *ρ*: correlation between outcome measurements; 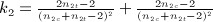; 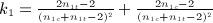; 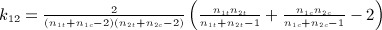.

### 3.5. Covariance between a standardized mean difference and a log odds ratio

Finally, we consider the covariance between an SMD (outcome 1) and a log odds ratio (outcome 2). The derivation proceeds in a similar way. Writing 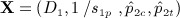, we presented the expectation of **X** in the previous two sections. In the covariance matrix, 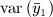 and var (1 / *s*_1*p*_) are given in Section 3.3, and 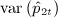 and 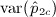 are given by (5). We show in Section A4 of our supplementary materials that


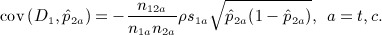
(13)

Implementing the bivariate delta method, we obtain


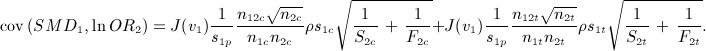


### 3.6. Generalization to other types of outcomes measures

In Table I, we present estimators for covariance for all combinations of mean difference, SMD, log odds ratio, log risk ratio, and risk difference. We derived the covariances involving log risk ratios and risk differences in the same way as those described previously. The computation of these covariances is straightforward if trial level summary statistics are reported. The formulae can also be used if individual patient data are available as an alternative to multivariate analysis of the raw data: we can extract the summary statistics in the formulae from the detailed data, and hence, we can derive the estimation of within-study covariances. This may be particularly attractive when dealing with a combination of continuous and dichotomous outcome data.

## 4. Practical issues

### 4.1. Simplifications to the formulae based

We have expressed the formulae for the most general case of knowing the sample sizes (say for the treatment group) of *n*_1*t*_, *n*_2*t*_, and *n*_12*t*_ for participants who report outcome 1, outcome 2, and both outcome 1 and outcome 2, respectively. Our formulae also allow for different standard deviations in different treatment groups when continuous data are involved. Some simplifications will often be made in practice. First consider the issue of sample sizes. In a study with no attrition, we have *n*_1*t*_ = *n*_2*t*_ = *n*_12*t*_ = *N*_*t*_ and *n*_1*c*_ = *n*_2*c*_ = *n*_12*c*_ = *N*_*c*_. Applying this, for instance, to the last covariance presented, between an SMD and a log odds ratio, we obtain


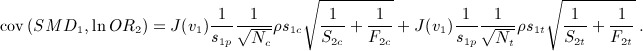


Often, however, different participants are missing values for different outcomes. In this case, we will typically not know the numbers *n*_12*t*_ and *n*_12*c*_ of participants who contribute to both outcomes. We can make various assumptions to approximate the covariance in this case. First, we could assume that those who report one outcome are a subset of those who report the other. Then, *n*_12*t*_ = min(*n*_1*t*_,*n*_2*t*_) and *n*_12*c*_ = min (*n*_1*c*_,*n*_2*c*_). The aforementioned covariance then simplifies to


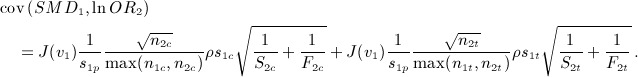


Second, we could assume that missingness of the two outcomes are independent, so that


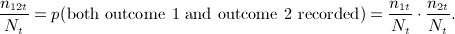


Hence, *n*_12*t*_ = *n*_1*t*_*n*_2*t*_ / *N*_*t*_ and *n*_12*c*_ = *n*_1*c*_*n*_2*c*_ / *N*_*c*_, yielding the simplification


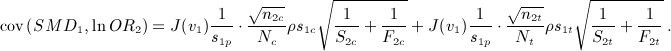


Participants known to contribute to neither outcome (e.g. early dropouts) may be omitted from the denominators *N*_*t*_ and *N*_*c*_.

It is often reasonable to assume that standard deviations are the same in the treatment and control groups, and this offers a further simplification to the covariance formulae. For instance, the previous covariance simplifies to


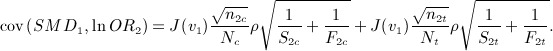


### 4.2. Positive semidefinite within-study variance matrix

Constraints on imputed between-outcome covariances need to be considered to ensure that the within-study covariance matrix **Σ** is semipositive definite. We suggest checking whether the eigenvalues of **Σ** are nonnegative. If this is the case, then **Σ** is semipositive definite; otherwise, the between-outcome covariances ought to be re-imputed with a different set of correlation coefficients. For the special case of two outcomes (bivariate meta-analysis), an alternative is simply to check that


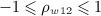
(14)

where *ρ*_*w*12_ are the correlations in **Σ**. For the case of three outcomes (trivariate meta-analysis), we can check that each correlation coefficient satisfies (14), and the determinant of **Σ** is nonnegative, that is, that


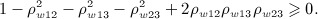
(15)

## 5. Simulation study

To further the proposed methodology, we conducted two simulation studies. Simulation studies from previous authors 9,16,19,29 have compared multivariate meta-analysis and univariate meta-analysis on the basis of aggregated data. Those simulations assume known within-study correlation between treatment effects.

### Comparison of methods

To investigate whether imputing correlations at the outcome level can improve the parameter estimates, we undertook a first simulation study with data simulated at the individual patient level. Section B of our supplementary material provided the simulation procedures and results for this study, and Supplementary Table B1 presented the characteristics of the simulations. We compare four approaches in a bivariate scenario with a continuous outcome and a dichotomous outcome. These approaches are: separate univariate meta-analyses, multivariate meta-analyses assuming zero within-study covariance, multivariate meta-analysis with a common within-study correlation between treatment effect estimates, and multivariate meta-analysis with a common within-study correlation between outcomes (which may lead to different covariances between treatment effects). Across 36 scenarios in which numbers of studies, numbers of participants, within-study correlations, between-study correlations, and between-study variances were varied, we observed very similar properties of treatment effect estimates across the four approaches. The two approaches that allowed for within-study covariance produced less biased estimates of the variance–covariance matrix, particularly when there is heterogeneity across studies for both outcomes. Our methods outperformed the approach that assumes the same covariance between treatment effects for every study, when the between-study correlation was low, but not otherwise. Between-study variance–covariance matrices were not well estimated when there only a few studies in the meta-analysis.

### Approximation error

In a second simulation study, we assessed approximation error in our formulae. In each investigation, we simulated correlated treatment effect estimates from 1000 studies using the same methods as in the first simulation (to obtain treatment effects for dichotomous outcomes, we dichotomized simulated normally distributed data). We computed the sample correlation coefficients between treatment effects for the 1000 studies to be used as a reference. We then approximated the within-study covariance for each study using relevant formulae in Table I. Dividing this by empirical standard deviations for the treatment effects, we obtained approximated within-study correlation coefficients. We then calculated the approximation error for each simulated study as the discrepancy between the approximated correlation and the sample correlation.

We repeated the procedures summarized above for 100 different correlation coefficients in the interval ( − 1,1). The Supplementary Figures B1–B3 give the mean squared errors from the simulations. We further assessed the dependence of approximation errors on sample sizes (distinguished by colors in these figures).

When both outcomes are dichotomous, our simulation shows that the formulae estimate the correlation coefficients with high accuracy, with mean squared errors very close to zero (less than 0.005). When one or both outcomes are continuous, the mean squared errors are bigger but less than 0.02. As noted in Section 3, the theoretical magnitude of the errors is partially affected by the squares of the outcome variables. It is thus expected that the errors are minimal when the effect sizes are small and larger when the effect sizes are large. Regarding dependency on sample size, mean squared errors are inflated when the number of participants is as small as 20, and the errors are less than 0.01 when sample size becomes greater. This suggests that the formulae are good approximation to the correlation coefficient and are most accurate for large studies. Future research on small-sample correction factors of the estimators will be useful.

## 6. Application to a meta-analysis of vasoactive drugs for acute stroke

To illustrate the use of the methods described in this paper, we consider a meta-analysis of placebo-controlled trials of vasoactive drugs for acute stroke 4. We take data from a selection of trials that contributed data on the four outcomes: systolic blood pressure (SBP, in mHg), diastolic blood pressure (DBP, in mHg), death (D), and ‘death or disability’ (DD). Supplementary Tables C1 and C2 give the raw data for this re-analysis. For trials with different sample sizes contributing to different outcomes, we take *n*_12*t*_ = min(*n*_1*t*_,*n*_2*t*_) and *n*_12*c*_ = min(*n*_1*c*_,*n*_2*c*_). We illustrate three analyses of these data, taking first the two continuous outcomes, second the two dichotomous outcomes, and then the full set of four outcomes. We implement all analyses (including univariate meta-analyses) using the Stata program *mvmeta* with restricted maximum likelihood (REML) estimation 30.

### 6.1. Bivariate meta-analysis of two correlated continuous outcomes

**Figure 1:**
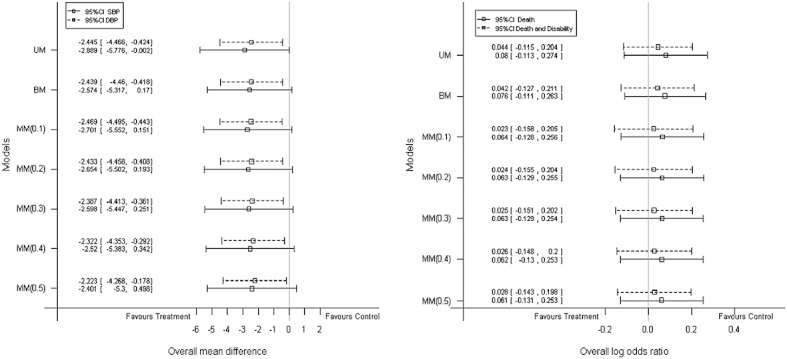
Estimated treatment effects and 95% confidence interval (CI) for systolic blood pressure (SBP; solid lines in left panel), diastolic blood pressure (DBP; dashed lines in left panel), death (D; solid lines in right panel), and death or disability (DD; dashed lines in right panel), based on separate univariate models (UM); bivariate model (BM) for SBP and DBP with covariance estimated from formula (2); bivariate model for D and DD with covariance estimated from formula (10); and multivariate model (MM) for all four outcomes, with imputed correlations (0.1,0.2,0.3,0.4,0.5) for remaining correlations.

We impute a correlation coefficient of 0.71 between SBP and DBP, on the basis of external evidence 31. We estimated the covariance between treatment effects on SBP and DBP using Equation (2); Supplementary Table C3 presented the values obtained for each study. Results of the bivariate meta-analysis are provided in Table II and illustrated in the bottom left of Figure 1 alongside results from standard univariate meta-analyses of the two outcomes separately. There is some evidence that SBP and DBP were lower in the treatment group than in the control group. However, the difference is under 3 mHg for both outcomes, which may not be clinically important. There is a slight increase in the precision of estimated treatment effects in the bivariate model than in the univariate model, and the amount of heterogeneity is reduced by a small amount for SBP. The correlation between treatment effects across studies is high at 0.973. Very high estimated correlations are a common finding in applications of multivariate meta-analysis: correlation is often estimated to be 1 or − 1 14.

**Table II:** Estimated treatment effects and standard errors from meta-analyses of systolic blood pressure (SBP) and diastolic blood pressure (DBP), based on univariate and bivariate random-effects models. We computes within-study correlation using Equation (2) with correlation of 0.71 assumed between SBP and DBP measurements 31

	Univariate meta-analyses	Bivariate meta-analysis
Mean difference in SBP	− 2.89 (1.47)	− 2.57 (1.40)
Mean difference in DBP	− 2.45 (1.03)	− 2.44 (1.03)
Heterogeneity SD for SBP	4.20	3.96
Heterogeneity SD for DBP	3.48	3.49
Correlation across studies	–	0.974

SD: standard deviation.

### 6.2. Bivariate meta-analysis of nested dichotomous outcomes

We can compute covariances between treatment effects for D and DD using Equation (10) without the need for knowledge of correlation between the two outcomes. Results are provided in Table III and illustrated in the bottom right of Figure 1. There is a lack of statistical evidence for treatment effects on D and DD. Again, we observe a slight increase in precision in the bivariate analysis and a high estimated between-study correlation. There is no discernable heterogeneity in either of these outcomes.

**Table III:** Estimated treatment effects and standard errors from meta-analyses of death (D) and ‘death or disability’ (DD), based on univariate and bivariate random-effects models. We computed within-study correlation using Equation (10)

	Univariate meta-analyses	Bivariate meta-analysis
Log OR for D	0.080 (0.099)	0.075 (0.098)
Log OR for DD	0.044 (0.096)	0.042 (0.095)
Heterogeneity SD for D	0.000	0.000
Heterogeneity SD for DD	0.000	0.000
Correlation across studies	–	0.969

OR: odds ratio; SD: standard deviation.

### 6.3. Multivariate meta-analysis of four outcomes

Some knowledge of the within-study correlations, either due to knowledge of correlations between outcomes or due to the nesting of one outcome within another, may motivate the aforementioned bivariate meta-analyses. We consider now the possibility of a multivariate meta-analysis of all four outcomes. Note that high-dimensional multivariate meta-analyses incorporate large numbers of parameters in the between-study covariance matrix and may involve more parameters than can reasonably be estimated from the data. However, we pursue this four-dimensional analysis to illustrate how a sensitivity analysis can be performed on the basis of between-outcome correlation coefficients.

**Figure 2:**
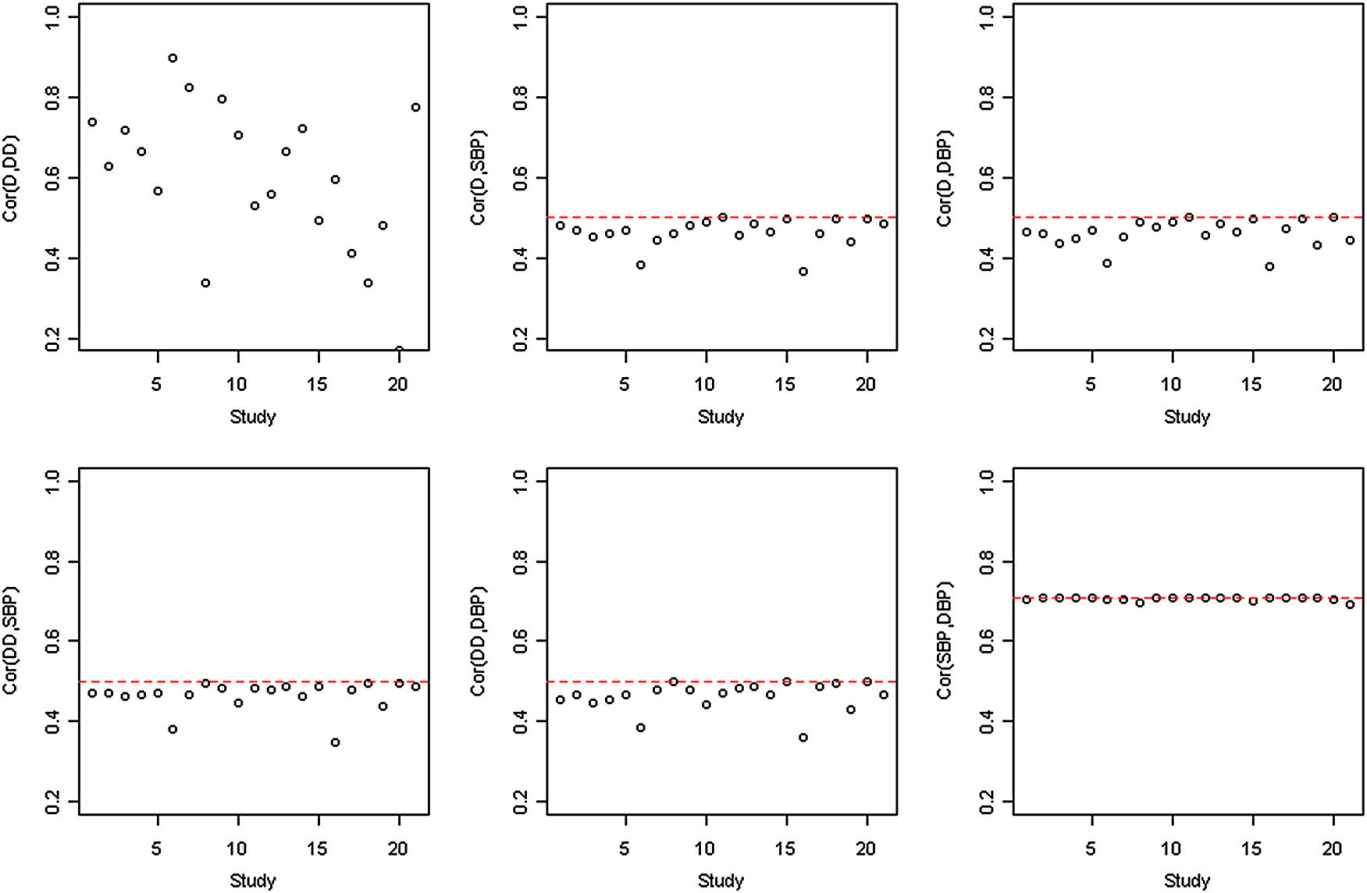
Approximated within-study between treatment effects correlation coefficients based on stroke data 4, from which we select 21 trials that reported all four outcomes. Horizontal dash lines denote imputed between-outcome correlations. Black circles denote approximated between treatment effects correlation. D, death; DD, death or disability; SBP, systolic blood pressure; DBP, diastolic blood pressure.

As in Section 6.1, we calculate covariances between SBP and DBP with Equation (2) by imputing a correlation coefficient of 0.71 31 and calculate the covariance between the two nested events D and DD with Equation (10). In the absence of information about correlation coefficients for the other pairs of outcomes, we impute them in five sensitivity analyses from a series of plausible values. We assume that high blood pressure is mildly positively correlated with death and with ‘death or disability’, and impute correlation coefficients between each pair of the continuous and dichotomous using values 0.1, 0.2, 0.3, 0.4, or 0.5. We then compute covariances between treatment effects for continuous and dichotomous outcomes using Equation (1.3) in Table I. Our formulae allow correlations between treatments effects to vary from one study to another, as illustrated in Figure 2. In this example, the correlation varies the most for the two nested events (D and DD) and the least for two mean differences (SBP and DBP).

We illustrate the overall effects from the four-dimensional multivariate analysis in Figure 1. The overall treatment effect estimates appear to be consistent across imputed correlation coefficients. Treatment effects for SBP and DBP change by less than 0.05, and those for D and DD range from 0.023 to 0.028 and from 0.061 to 0.064, respectively, on the log odds ratio scale. Between-study variance estimates are also insensitive to the varying imputed correlations.

**Figure 3:**
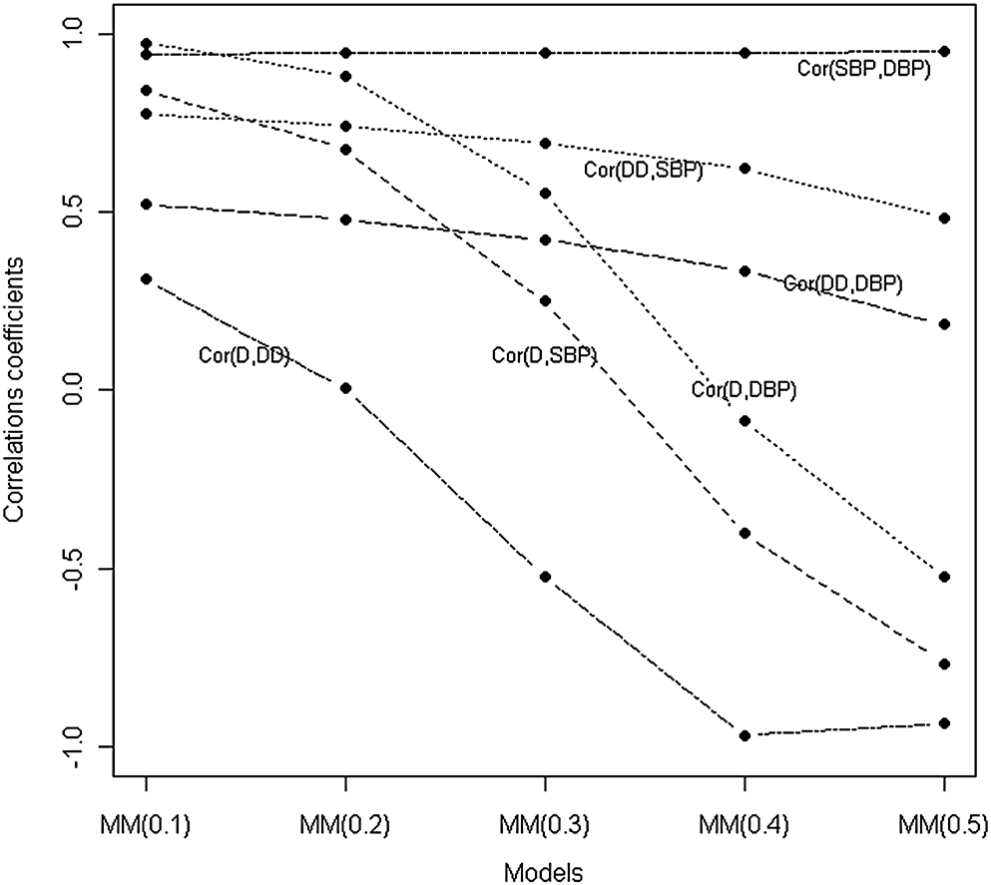
Estimated between-study correlation coefficients, based on four-dimensional multivariate meta-analysis model (MM) with imputed correlation 0.71 between systolic blood pressure (SBP) and systolic blood pressure (DBP), and various values (0.1,0.2,0.3,0.4,0.5) for correlations between SBP and death (D) or death or disability (DD), and DBP and D or DD.

However, between-study correlations are affected in the sensitivity analysis. Figure 3 illustrates estimates of between-study correlation for the five sensitivity analyses. Correlations between SBP and DBP are consistently above 0.9, but the between-study correlation estimates for other pairs of outcomes are substantially different in different sensitivity analyses. Between-study correlations change from high positive correlation to high negative correlation as within-study correlations increase from 0.1 to 0.5.

## 7. Discussion

Multivariate meta-analysis can offer advantages over a univariate approach, particularly when there are nonignorable missing data 8,9,16. Multivariate meta-analysis of multiple outcomes requires specification of within-study covariances. We have derived approximate formulae for such within-study covariances for various pairs of treatment effect measures for situations in which individual patient data are not available but correlations between outcomes can be specified. Our derivations are motivated by the notion that correlations among outcome measures are often well understood. Nevertheless, sensitivity analyses should typically accompany imputation of such values from external sources. Our simulation studies assess whether imputing correlation at the outcome level can improve estimation compared with alternative approaches. We conclude that when there is heterogeneity of effects across studies and high correlation within studies, our approach outperforms others when there are complete data.

We have taken into account within-study missing data. Our formulae allow for different numbers of participants to contribute to different outcomes. Not all participants randomized in a trial will necessarily report on all outcomes, for example if different outcomes are measured at different time points in a trial that suffers from attrition over time. The formulae simplify if the same participants can be assumed to contribute to both outcomes.

We have considered both continuous and dichotomous outcomes, which are the most common types of data in meta-analysis. We could derive covariances for other types of data in similar ways. Most of the formulae were based on a bivariate delta method and so will be most accurate for large studies. This was confirmed by our simulation studies, which found that approximation errors, although small in general, were even smaller in large studies (more than 20 participants). An alternative approach is to place prior distributions on correlation coefficients within a Bayesian framework. However, readily available Bayesian approaches to multivariate meta-analysis are currently restricted to two outcome problems. Addressing multiple outcomes introduces complications in trying to ensure a positive semidefinite constraint on the variance–covariance matrix 23.

We have focused on within-study covariance estimation. However, estimation of the between-study covariance matrix is a substantial problem that has been noted before 14 and is illustrated in our application. The estimation problem is not unique to meta-analysis but a common problem in classical multivariate analysis. When the number of outcomes, *p*, is large relative to the number of studies, *n*, estimation of the covariance matrix can be difficult unless *p* / *n* is small 32,33. In multivariate meta-analysis with multiple outcomes, the number of outcomes *p* can easily become very large compared with the number of studies, which is typically rather small in meta-analysis. We are unable to provide specific guidance on how large *n* needs to be for reliable estimation of *p* outcomes in a multivariate meta-analysis. More comprehensive simulation studies might be undertaken to examine this question. Future research is also required to seek improved estimation of between-study covariance parameters or to determine how model complexity can be reduced.

## Footnotes

‡ Supporting information may be found in the online version of this article.
