# Supplementary material for: Estimating within-study covariances in multivariate meta-analysis with multiple outcomes
Source: Stat Med. 2012 Dec 3;32(7):1191–205. doi: 10.1002/sim.5679 (PMC3618374; doi:10.1002/sim.5679)
Supplement: Supplementary file 1 [file sim0032-1191-SD1.pdf]

Supplementary materials for "Estimating within-study covariances in multivariate meta-analysis with multiple outcomes"

YINGHUI WEI[*], JULIAN PT HIGGINS

SUPPLEMENTARY MATERIAL A

DERIVATION OF COVARIANCES BETWEEN PARTICULAR PAIRS OF SUMMARY STATISTICS

A1. Derivation of equation (6)

We first derive equation (6), the covariance between two sample probabilities for two outcomes within a treatment group. Let $I_{c1i}, i = 1, 2, ..., n_{1c}$ and $I_{c2i}, i = 1, 2, ..., n_{2c}$ denote Bernoulli variables, which take value 1 for the event of interest with probabilities p_{1c} and p_{2c} and value 0 otherwise. Given knowledge of the correlation between the dichotomous variables, i.e. $\rho = corr(I_{c1i}, I_{c2i})$, we can deduce that

$$\begin{aligned}
\text{cov}(\hat{p}_{1c}, \hat{p}_{2c}) &= \text{cov}\left(\frac{S_{1c}}{n_{1c}}, \frac{S_{2c}}{n_{2c}}\right) \\
&= \frac{1}{n_{1c}n_{2c}} \text{cov}(S_{1c}, S_{2c}) \\
&= \frac{1}{n_{1c}n_{2c}} \text{cov}\left(\sum_{i=1}^{n_{1c}} I_{1ci}, \sum_{i=1}^{n_{2c}} I_{2ci}\right) \\
&= \frac{1}{n_{1c}n_{2c}} \cdot \sum_{i=i'} \text{cov}(I_{1ci}, I_{2ci'}) \\
&= \frac{n_{12c}}{n_{1c}n_{2c}} \rho \sqrt{p_{1c}(1-p_{1c})p_{2c}(1-p_{2c})}
\end{aligned}$$

and similarly

[*] To whom correspondence should be addressed. y.wei@ctu.mrc.ac.uk

$$\text{cov}(\hat{p}_{1t}, \hat{p}_{2t}) = \frac{n_{12t}}{n_{1t}n_{2t}} \rho \sqrt{p_{1t}(1-p_{1t})p_{2t}(1-p_{2t})} \ .$$

A2. Derivation of equation (9)

We now derive equation (9), the covariance term between probabilities for two outcomes, given that outcome 1 is nested within outcome 2. We illustrate the covariance term for the treatment group; the covariance term for the control group can be derived in a similar way.

Suppose we observe S_{1t} out of N_t participants with outcome 1, and S_{2t} out of N_t participants with outcome 2. Since outcome 1 is nested within outcome 2, we have $S_{2t} = S_{1t} + a_t \ \ (a_t \geq 0)$ and $p_{2t} = p_{1t} + p_{at}$, with p_{1t}, p_{2t} and p_{at} being the probabilities of events S_{1t}, S_{2t} and a_t respectively. We assume a multinomial distribution: $(S_{1t}, a_t) \sim multinomial(N_t, p_{1t}, p_{at})$. Then

$$\begin{aligned}
\text{cov}(p_{1t}, p_{2t}) &= \text{cov}\left(\frac{S_{1t}}{N_t}, \frac{S_{2t}+a_t}{N_t}\right) \\
&= \text{cov}\left(\frac{S_{1t}}{N_t}, \frac{S_{1t}+a_t}{N_t}\right) \\
&= \frac{1}{N_t^2}\text{cov}(S_{1t}, S_{1t}+a_t) \\
&= \frac{1}{N_t^2}\text{var}(S_{1t}) + \frac{1}{N_t^2}\text{cov}(S_{1t}, a_t) \\
&= \frac{1}{N_t} p_{1t}(1-p_{1t}) - \frac{1}{N_t} p_{1t}p_{at}
\end{aligned}$$

In practice, we might observe different denominators (n_{1t}, n_{2t}) for outcomes 1 and 2. Typically this situation will be associated with $n_{2t} < n_{1t}$, since information on event ‘A or B’ should more readily be available than information on event ‘A alone’. Thus we take

$N_t = n_{1t}$. For the $n_{1t} - n_{2t}$ participants for whom we do not know their outcomes, we assume they are missing at random and assign a proportion $\left(S_{2t} - S_{1t}\right)/\left(n_{2t} - S_{1t}\right)$ of them to event ‘B not A’ (equivalently ‘A or B’) and the remainder to event ‘neither A nor B’. This yields a revised value of a_t, say $a_t' = a_t + (n_{1t} - n_{2t}) \times \dfrac{S_{2t} - S_{1t}}{n_{2t} - S_{1t}}$.

A3 Derivation of equation (12)

Now we derive equation (12), the covariance between two inverse sample variances for two continuous outcomes within a study. We use the properties of the bivariate chi-squared distribution to assist this deviation.

Bivariate chi-squared distribution

Let (Z_1, Z_2) be a two dimensional correlated random vector with mean $(\mu_1, \mu_2)^T$ and variance-covariance matrix $\mathbf{\Sigma}$ having elements σ_{ik} for i = 1, 2; k = 1, 2, where $\sigma_{11} = \sigma_1^2$, $\sigma_{22} = \sigma_2^2$, $\sigma_{12} = \rho\sigma_1\sigma_2$, and ρ is a correlation coefficient. Let s_1^2 and s_2^2 be sample variances for realizations of Z_1 and Z_2. Then random quantities $U = vs_1^2/\sigma_1^2$ and $V = vs_2^2/\sigma_2^2$ follow a bivariate chi-squared distribution with probability density function described by Joarder (2007; 2008a; 2008b). Two useful properties are:

Property 1: $\text{corr}(U,V) = \dfrac{\text{cov}(U,V)}{\sqrt{\text{var}(U)\,\text{var}(V)}} = \rho^2$

Property 2: $\text{var}(U) = \text{var}(V) = 2v$

Now consider the situation of two outcomes being measured in each of two treatment arms. We use s_{ja}^2 to denote the sample variance for outcome j = 1 or 2 in treatment arm a = t or c. The pooled sample variance can be written as

$$s_{jp}^2 = \lambda_{jt} \cdot s_{jt}^2 + \lambda_{jc} \cdot s_{jc}^2$$

where $\lambda_{ja} = v_{ja} \big/ \left(n_{jc} + n_{jt} - 2\right)$, with $v_{ja} = n_{ja} - 1$ being the degrees of freedom for outcome j in treatment arm a. The variances of the pooled sample variances are

$$\begin{aligned}
\text{var}(s_{1p}^2) &= \text{var}(\lambda_{1t}s_{1t}^2 + \lambda_{1c}s_{1c}^2) \\
&= \lambda_{1t}^2 \,\text{var}(s_{1t}^2) + \lambda_{1c}^2 \,\text{var}(s_{1c}^2) \\
&= 2\lambda_{1t}^2\sigma_1^4 v_{1t}^{-1} + 2\lambda_{1c}^2\sigma_1^4 v_{1c}^{-1} \\
&= 2(\lambda_{1t}^2 v_{1t}^{-1} + \lambda_{1c}^2 v_{1c}^{-1})\sigma_1^4 \\
&= 2(\lambda_t^2 v_t^{-1} + \lambda_c^2 v_c^{-1})\sigma_1^4 \qquad (\text{if } n_{1t} = n_{2t} \text{ and } n_{1c} = n_{2c})
\end{aligned}$$

and

$$\text{var}(s_{2p}^2) = 2(\lambda_t^2 v_t^{-1} + \lambda_c^2 v_c^{-1})\sigma_2^4 \qquad (\text{if } n_{1t} = n_{2t} \text{ and } n_{1c} = n_{2c})$$

To obtain the covariances, we first apply Property 1 to the sample variances:

$$\begin{aligned}
corr(s_{1a}^2, s_{2a}^2) &= \frac{\text{cov}(s_{1a}^2, s_{2a}^2)}{\sqrt{\text{var}(s_{1a}^2)\,\text{var}(s_{2a}^2)}} \\
&= \frac{\text{cov}(U\sigma_1^2/v_{1a}, U\sigma_2^2/v_{2a})}{\sqrt{\text{var}(U\sigma_1^2/v_{1a})\,\text{var}(U\sigma_2^2/v_{2a})}} \\
&= \frac{\text{cov}(U, V)}{\sqrt{\text{var}(U)\,\text{var}(V)}} \\
&= \rho^2
\end{aligned}$$

and then derive

$$
\begin{aligned}
\operatorname{cov}(s_{1p}^2, s_{2p}^2) &= \operatorname{cov}(\lambda_{1t}s_{1t}^2 + \lambda_{1c}s_{1c}^2, \lambda_{2t}s_{2t}^2 + \lambda_{2c}s_{2c}^2) \\
&= \lambda_{1t}\lambda_{2t}\operatorname{cov}(s_{1t}^2, s_{2t}^2) + \lambda_{1c}\lambda_{2c}\operatorname{cov}(s_{1c}^2, s_{2c}^2) \\
&= \lambda_{1t}\lambda_{2t}\cdot 2\rho^2 v_{12t}\cdot \sigma_1^2 v_{12t}^{-1}\sigma_2^2 v_{12t}^{-1} + \lambda_{1c}\lambda_{2c}\cdot 2\rho^2 v_{12c}\cdot \sigma_1^2 v_{12c}^{-1}\sigma_2^2 v_{12c}^{-1} \\
&= 2\lambda_{1t}\lambda_{2t}v_{12t}^{-1}\rho^2\sigma_1^2\sigma_2^2 + 2\lambda_{1c}\lambda_{2c}v_{12c}^{-1}\rho^2\sigma_1^2\sigma_2^2 \\
&= 2(\lambda_{1t}\lambda_{2t}v_{12t}^{-1} + \lambda_{1c}\lambda_{2c}v_{12c}^{-1})\rho^2\sigma_1^2\sigma_2^2
\end{aligned}
$$

Let $k_1 = 2(\lambda_{1t}^2 v_{1t}^{-1} + \lambda_{1c}^2 v_{1c}^{-1})$, $k_2 = 2(\lambda_{2t}^2 v_{2t}^{-1} + \lambda_{2c}^2 v_{2c}^{-1})$, and $k_{12} = 2(\lambda_{1t}\lambda_{2t}v_{12t}^{-1} + \lambda_{1c}\lambda_{2c}v_{12c}^{-1})$. Then the covariance matrix for s_{1p}^2 and s_{2p}^2 is given by

$$
\mathbf{\Sigma}^* = \begin{bmatrix} k_1\sigma_1^4 & k_{12}\rho^2\sigma_1^2\sigma_2^2 \\ k_{12}\rho^2\sigma_1^2\sigma_2^2 & k_2\sigma_2^4 \end{bmatrix}.
$$

We derive the desired $\operatorname{cov}\left(s_{1p}^{-1}, s_{2p}^{-1}\right)$ using the delta method as described in the main text.

Writing $\mathbf{X} = (s_{1p}^2, s_{2p}^2)$ we take $f_1(\mathbf{X}) = s_{1p}^{-1}$ and $f_2(\mathbf{X}) = s_{2p}^{-1}$, then

$$
\nabla f^*(\mathbf{X}) = \begin{bmatrix} -\frac{1}{2}s_{1p}^{-\frac{3}{2}} & 0 \\ 0 & -\frac{1}{2}s_{1p}^{-\frac{3}{2}} \end{bmatrix},
$$

$$\begin{aligned}\mathbf{\Sigma}^{**} &= \nabla f^{*T}\mathbf{\Sigma}^{*}\nabla f^{*} \\ &= \begin{bmatrix} -\frac{1}{2}\sigma_1^{-3} & 0 \\ 0 & -\frac{1}{2}\sigma_2^{-3} \end{bmatrix} \mathbf{\Sigma}^{*} \begin{bmatrix} -\frac{1}{2}\sigma_1^{-3} & 0 \\ 0 & -\frac{1}{2}\sigma_2^{-3} \end{bmatrix} \\ &= \begin{bmatrix} \frac{1}{4}k_1\sigma_1^{-2} & \frac{1}{4}k_{12}\rho^2\sigma_1^{-1}\sigma_2^{-1} \\ \frac{1}{4}k_{12}\rho^2\sigma_1^{-1}\sigma_2^{-1} & \frac{1}{4}k_2\sigma_2^{-2} \end{bmatrix}\end{aligned}$$

That is

$$corr(s_{1p}^{-1}, s_{2p}^{-1}) = \frac{k_{12}}{\sqrt{k_1k_2}}\rho^2 .$$

Therefore, by using the approximate variance for s_{1p}^{-1} and s_{2p}^{-1}, we have

$$\begin{aligned}\operatorname{cov}(s_{1p}^{-1}, s_{2p}^{-1}) &= \frac{k_{12}}{\sqrt{k_1k_2}}\rho^2\sqrt{\operatorname{var}(s_{1p}^{-2})\operatorname{var}(s_{2p}^{-2})} \\ &= \frac{k_{12}}{\sqrt{k_1k_2}}\rho^2\frac{1}{\sigma_1\sigma_2}\sqrt{\left(\frac{v_1}{v_1-2}-\frac{1}{J(v_1)^2}\right)\left(\frac{v_2}{v_2-2}-\frac{1}{J(v_2)^2}\right)}.\end{aligned}$$

A4. Derivation of equation (13)

Finally we derive equation (13) the covariance between an estimated mean difference and a sample probability.

$$\begin{aligned}\operatorname{cov}\left(\bar{y}_{1c}-\bar{y}_{1t},\hat{p}_{2c}\right)&=\operatorname{cov}\left(\bar{y}_{1c},\hat{p}_{2c}\right)\\&=\operatorname{cov}\left(\bar{y}_{1c},\frac{S_{2c}}{n_{2c}}\right)\\&=\operatorname{cov}\left(\frac{1}{n_{1c}}\sum_{i=1}^{n_{1c}}y_{1ci},\frac{1}{n_{2c}}\sum_{i=1}^{n_{2c}}I_{2ci}\right)\\&=\frac{1}{n_{1c}n_{2c}}\sum_{i=i'}\operatorname{cov}\left(y_{1ci},I_{2ci'}\right)\\&=\frac{n_{12c}}{n_{1c}n_{2c}}\rho s_{1c}\sqrt{p_{2c}(1-p_{2c})}\end{aligned}$$

since I_{2ci} follows a Bernoulli distribution. Similarly,

$$\operatorname{cov}\left(\bar{y}_{1c}-\bar{y}_{1t},\hat{p}_{2t}\right)\qquad=\frac{n_{12t}}{n_{1t}n_{2t}}\rho s_{1t}\sqrt{p_{2t}(1-p_{2t})}\quad .$$

REFERENCES FOR SUPPLEMENTARY MATERIAL A

Joarder AH. A bivariate chi-square distribution and some of its properties. KFUPM, Department of Mathematics & Statistics, *Technical report*, 2007.

Joarder AH, Abujiya MR. Standardized moments for bivariate chi-square distribution. *Journal of Applied Statistical Science* 2008a; **16**:1-9.

Joarder AH and Omar MH. Some Statistical Properties of a Bivariate Chi-Square Probability Density Function. KFUPM, Department of Mathematics & Statistics, *Technical report* 382, 2008b.

SUPPLEMENTARY MATERIAL B

B1 Simulation procedures

Overview We simulate meta-analysis data for two outcomes, with outcome 1 being a continuous variable and outcome 2 being a dichotomous variable. The treatment effects are measured using a mean difference and a log odds ratio for the two outcomes respectively. To induce correlation between the outcomes within studies, we simulate individual participant outcomes from a bivariate normal distribution and dichotomize the second variable. The correlations between the continuous and dichotomous outcomes, and between the treatment effect estimates for the two outcomes, are estimated empirically.

Simulation parameter specification We consider a wide range of sample sizes to assess the dependence of estimation properties on the number of studies (N_s), the number of participants in treatment (N_t) and control groups (N_c), as well as the degree of dependence between outcomes. We approximately mimic the SBP and DBP outcomes in the acute stroke data by setting the between-study parameters as

$$\theta_1 = -2.58,\ \theta_2 = 0.06,\ \tau_1^2 = 4.20^2,\ \tau_2^2 = 0.000,$$

and within-study (between-participant) variances as

$$\sigma_1^2 = 20^2,\ \sigma_2^2 = 0.5\,.$$

To further evaluate the impact of the heterogeneity on the parameters estimates, we extend the simulation to consider $\tau_2^2 = 0.1^2$ and $\tau_2^2 = 0.2^2$, while keeping the other parameters unchanged. We evaluate combinations of within-study correlation (ρ_w) and

between-study correlation (ρ_b), setting each to be either zero or strong (0.9). We perform simulations under a scenario in which the within-study treatment effects are the same for every study, and for a scenario in which within-study treatment effect variances vary across studies. We achieve the latter by keeping the between-participant outcome variances the same, and varying the sample sizes within studies. The full set of scenarios considered is provided in Supplementary Table B1.

Outcome data generation We start by simulating true treatment effects for two continuous outcomes using a bivariate normal distribution:

$$\begin{pmatrix}\theta_{1s}\\ \theta_{2s}\end{pmatrix} \sim MVN\left(\begin{pmatrix}\theta_1\\ \theta_2\end{pmatrix}, \Omega\right) \text{ with } \Omega = \begin{bmatrix}\tau_{11}^2 & \rho_b\tau_{11}\tau_{22}\\ \rho_b\tau_{11}\tau_{22} & \tau_{22}^2\end{bmatrix}.$$

with parameter values as specified above. Within each study, we simulate individual outcome data for each participant *i*, centering the control group participants on the observed control group mean across acute stroke trials for outcome 1 (the means and standard deviations for outcome 2 are arbitrary). The treatment group means are obtained by applying the simulated treatment effect for the study:

$$\begin{pmatrix}y_{1csi}\\ y_{2csi}\end{pmatrix} \sim MVN\left(\begin{pmatrix}\mu_{1c}\\ \mu_{2c}\end{pmatrix}, \mathbf{\Sigma}\right), \begin{pmatrix}y_{1tsi}\\ y_{2tsi}\end{pmatrix} \sim MVN\left(\begin{pmatrix}\mu_{1c}+\theta_{1s}\\ \mu_{2c}+\theta_{2s}\end{pmatrix}, \mathbf{\Sigma}\right) \text{ with } \mathbf{\Sigma} = \begin{bmatrix}\sigma_1^2 & \rho_w\sigma_1\sigma_2\\ \rho_w\sigma_1\sigma_2 & \sigma_2^2\end{bmatrix}.$$

We then dichotomize each y_{2tsi} and y_{2csi} using cut-points c_t and c_c which are chosen so that $\Pr(y_{2tsi} \geq c_t) = p_t$ and $\Pr(y_{2csi} \geq c_c) = p_c$ with p_t and p_c the observed overall probabilities of death in the acute stroke data. These cutpoints define true odds ratios that are the same in every study, reflecting the lack of heterogeneity observed in the acute

stroke data. The numbers of observations lying beyond the cut point are given by r_{ts} and r_{cs} in the two arms.

Effect size calculation We calculate the effect sizes and the associated variances for study *s* by

$$MD_s = \bar{y}_{1ts} - \bar{y}_{1cs}, \quad \mathrm{var}(MD_s) = \left(\frac{1}{N_t} + \frac{1}{N_c}\right)\sigma_1^2$$

$$\ln OR_s = \ln\left(\frac{r_{ts} / (N_t - r_{ts})}{r_{cs} / (N_c - r_{cs})}\right), \quad \mathrm{var}(\ln OR_s) = \frac{1}{r_{ts}} + \frac{1}{N_t - r_{ts}} + \frac{1}{r_{cs}} + \frac{1}{N_c - r_{cs}}$$

for mean difference and log odds ratio, respectively.

Fitted models We fit four models to the simulated data.

MU [Univariate meta-analyses]: Standard univariate meta-analyses are performed for the two outcomes separately.

MM(0) [Multivariate meta-analysis assuming zero within-study correlation): Within-study correlation, ρ_w, is assumed to be zero.

MM(ρ_e) [Multivariate meta-analysis with a common within-study covariance for treatment effects for every study]: We calculate the within-study covariance between effect sizes as

$$\mathrm{cov}(\mathrm{MD}, \log OR) = \rho_e \sqrt{\mathrm{var}(MD)\,\mathrm{var}(\log OR)}\,.$$

MM(ρ_e) [Multivariate meta-analysis with within-study covariances estimated from between-outcome correlations based on our formulae]: We calculate within-study covariance as

$$\text{cov}(\text{MD}, \log OR) = \rho_o \sigma_1 \frac{1}{\sqrt{n_c}} \sqrt{\frac{1}{r_c} + \frac{1}{n_c - r_c}} + \rho_o \sigma_1 \frac{1}{\sqrt{n_t}} \sqrt{\frac{1}{r_t} + \frac{1}{n_t - r_t}}$$

as in formula (1.3) of Table 1.

For the latter two methods, both correlation between effect sizes ρ_e and correlation between outcomes ρ_o are obtained from a pre-simulation run, and are kept fixed in the estimation. Correlation between outcome ρ_o is invariant to sample size (number of participants in a study), while ρ_e can vary with sample size (see Supplementary Table B1).

Estimation process We report the average bias for the estimated treatment effects $\hat{\theta}_1 = MD$ and $\hat{\theta}_2 = \ln OR$ and the elements of the variance-covariance matrix for these $\hat{\tau}_{11}^2$, $\hat{\tau}_{22}^2$ and $\hat{\tau}_{12}$ over 1000 Monte Carlo replications. We calculate the 95% confidence interval (CI) of $\hat{\theta}_i$ for the two outcomes using using $\hat{\theta}_j \pm t_{0.025,(n-1)} \sqrt{\text{var}(\hat{\theta}_j)};\ j = 1, 2$ where $t_{0.025,(n-1)}$ denote the 0.025 percentile of a *t*-distribution with $n-1$ degrees of freedom, with n the total number of studies. This t-distribution is commonly used in the meta-analysis literature (Riley *et al.* 2007a, 2007b; Jackson *et al.* 2011; Ma & Mazumda 2011), although it is only an approximation (Riley *et al.* 2007b). We also calculated the proportion of the 95% CIs that cover the true θ_j *j=1,2*. We estimated the mean squared error $\text{MSE}(\hat{\theta}_j)$ across the 1000 simulations as $\sum_{k=1}^{1000} \left(\hat{\theta}_{jk} - \theta_j\right)^2 / 1000$. We carried out all data simulations and analyses using the software R (R Foundation for Statistical Computing, Vienna, Austria).

Results Summary statistics from the simulations are given in Supplementary Table B2 – B4. Our first observation is that treatment effects are well estimated by all methods, and there is little difference between univariate and multivariate approaches. We note that multivariate meta-analysis is most likely to offer advantages over a univariate approach when there are non-ignorable missing data (Kirkham *et al*. 2012). Our simulation study used complete case data and did not address this issue.

Comparing results across Tables B2-B4 shows the impact of the extent of heterogeneity for outcome two, with $\tau_2^2 = 0, 0.1^2, 0.2^2$, respectively. Table B2 shows that when $\tau_2^2 = 0$, the multivariate approach reduces bias in estimating τ_1 but increase bias for τ_2. It is therefore not clear whether a multivariate approach is better than a univariate approach when there is heterogeneity in one outcome but not in the other. However, when τ_2 slightly departs from zero, improvement in estimates for between-study variance is evident in scenario 13-16,18,21-22 in Table B3. If we increase the τ_2 to 0.2^2, there are consistent improvements in parameters estimates for between-study variance for both outcomes through all scenarios in Table B4. This suggests that a multivariate approach is likely to outperform a univariate approach when there is heterogeneity for both outcomes, but not necessarily otherwise.

We turn now to the impact of the magnitude of the correlation. Strong within-study correlation is assumed in scenarios 13-20 and 25-32. In these situations, both UM and MM(0) are misspecified models with respect to the within-study correlation. MM(ρ_e)

assumes a common correlation between treatment effects and MM(ρ_o) assumes a common correlation between outcomes, using our formulae to approximate the within-study covariance. We expect that UM and MM(0) will be worse than the latter two with misspecification of the within-study correlation; while MM(ρ_o) should be similar to MM(ρ_e). We observe that bias for τ_{11}^2 in UM and bias for τ_{22}^2 in MM(0) are inflated in scenarios 13-20 and 25-32. In Table B3, the bias for between-study correlation ρ_b in MM(0) is sometimes inflated, particularly in scenarios 13-14, and 17-18 where the between-study correlation is zero; the biases are 0.78, 0.92, 0.85 and 0.66, respectively. These biases appear to be serious, given the parameter space [-1, 1] for correlation coefficients. Similarly findings are given in scenarios 25-26, and 29-30. These indicate that when correlation is strong (weak) for within- (between-) study, then assuming zero within-study correlation will introduce bias into estimates for between-study correlation. This is one other situation in which correct specification or approximation of within-study correlation is important.

Estimation from MM(ρ_o) is generally not worse than MM(ρ_e). The particular situation in which we expect MM(ρ_o) to perform better is when sample sizes vary across studies (the even-numbered scenarios), since the covariances between treatment effects then vary across studies even if the covariances between outcomes remain the same. We observe such a pattern for situations in which between-study correlation is low, but not when between-study correlation is high (which might be explained in part by our relatively

small underlying heterogeneity variance for the second outcome, which does not allow the high between-study correlation to manifest itself).

Finally, Tables B3 and B4 show that, if there are large number of studies (n=50), the bias in the between-study variance estimate is reduced by using a multivariate approach, and the bias in the between-study correlation estimate is minimized in our proposed approach. However, when there are a few studies (n=10), no clear improvement are observed in the multivariate approach over the univariate approach, unless the within- (between-) study correlations are high (low).

REFERENCES FOR SUPPLEMENTARY MATERIAL B

Jackson D, White IR, Thompson SG. Extending DerSimonian and Laird's methodology to perform multivariate random effects meta-analyses. *Stat.Med.* 2010; **29**(12):1282-1297.

Riley RD, Abrams KR, Lambert PC, Sutton AJ, Thompson JR. An evaluation of bivariate random-effects meta-analysis for the joint synthesis of two correlated outcomes. *Stat.Med.* 2007; **26**(1):78-97.

Riley RD, Abrams KR, Sutton AJ, Lambert PC, Thompson JR. Bivariate random-effects meta-analysis and the estimation of between-study correlation. *BMC.Med.Res.Methodol.* 2007; **7**:3.

Ma Y, Mazumdar M. Multivariate meta-analysis: a robust approach based on the theory of U-statistic. *Stat.Med.* 2011; **30**(24):2911-2929.

Kirkham JJ, Riley RD, Williamson PR. A multivariate meta-analysis approach for reducing the impact of outcome reporting bias in systematic reviews. *Stat.Med.* 2012; **31**(20):2179-2195.

Supplementary Table B1 Correlation between effect size and outcomes based on data simulated from bivariate normal distribution with within- and between study correlation ρ_w and ρ_b. Scenario numbers appear in square brackets.

Pre-specified ρ_w	Pre-specified ρ_b	ρ_o based on 1000 Monte-Carlo simulation	ρ_e based on variable sample size			
			$n_s = 50$		$n_s = 10$	
			$n_t(n_c) = 200$	U (20,200)	$n_t(n_c) = 20$	U (20,200)
0.9	0	0.667	0.30 [1]	0.37 [2]	0.45 [5]	0.27 [6]
0.9	0.9	0.668	0.52 [3]	0.55 [4]	0.50 [7]	0.59 [8]
0	0	0.002	0.05 [9]	-	-0.03 [11]	-
0	0.9	0.016	0.26 [10]	-	0.032 [12]	-

ρ_b = between-study correlations; ρ_w = within-study correlations; ρ_o = Estimated within-study correlations between outcomes; ρ_e = Estimated within-study correlations between treatment effects, given sample sizes; n_s =Number of studies; $n_t(n_c)$ = Number of participants randomized in treatment (control) groups.

Supplementary Table B2. Simulation results over 1000 Monte Carlo replications.

True values for between-study parameters $\beta_1 = -2.58$, $\beta_2 = 0.06$, $\tau_1^2 = 4.2^2$, $\tau_2^2 = 0.000$. Within-study parameters $\sigma_1^2 = 20^2$, $\sigma_2^2 = 0.5$, $p_t = 0.197$(probability in treatment group), $p_c = 0.192$(probability in control group).

Methods	Bias-M $\hat{\beta}_1$	Bias-M $\hat{\beta}_2$	MSE $\hat{\beta}_1$	MSE $\hat{\beta}_2$	t coverage $\hat{\beta}_1$	t coverage $\hat{\beta}_2$	Bias-M $\hat{\tau}_1$	Bias-M $\hat{\tau}_2$	Bias-M $\hat{\rho}_{12}$
Scenario 1: $n_s = 50$, $n_t = n_c = 200$, $\rho_w = 0.9$, $\rho_b = 0$.									
UM	0.009	-0.009	0.450	0.001	0.949	0.943	-0.016	0.020	NA
MM(0)	0.009	-0.009	0.450	0.001	0.949	0.947	-0.014	0.059	0.887
MM(ρ_e)	0.009	-0.009	0.450	0.001	0.949	0.944	-0.014	0.043	0.634
MM(ρ_o)	0.008	-0.009	0.450	0.001	0.949	0.947	0.003	0.035	0.005
Scenario 2: $n_s = 50$, $n_t = n_c \sim U(20,\ 200)$, $\rho_w = 0.9$, $\rho_b = 0$.									
UM	-0.009	-0.013	0.504	0.002	0.944	0.947	-0.046	0.025	NA
MM(0)	-0.009	-0.012	0.509	0.002	0.947	0.955	-0.012	0.097	0.953
MM(ρ_e)	-0.010	-0.013	0.500	0.002	0.940	0.948	-0.053	0.057	0.631
MM(ρ_o)	-0.010	-0.013	0.498	0.002	0.945	0.949	-0.012	0.046	0.101
Scenario 3: $n_s = 50$, $n_t = n_c = 200$, $\rho_w = 0.9$, $\rho_b = 0.9$.									
UM	0.009	-0.009	0.450	0.001	0.949	0.944	-0.016	0.020	NA
MM(0)	0.009	-0.009	0.450	0.001	0.949	0.949	-0.013	0.068	0.041
MM(ρ_e)	0.009	-0.009	0.450	0.001	0.949	0.948	-0.014	0.048	-0.143
MM(ρ_o)	0.009	-0.009	0.450	0.001	0.949	0.947	0.002	0.035	-0.595
Scenario 4: $n_s = 50$, $n_t = n_c \sim U(20,\ 200)$, $\rho_w = 0.9$, $\rho_b = 0.9$.									
UM	-0.009	-0.013	0.504	0.002	0.944	0.946	-0.046	0.026	NA
MM(0)	-0.009	-0.012	0.509	0.002	0.948	0.959	-0.006	0.107	0.075
MM(ρ_e)	-0.009	-0.012	0.500	0.002	0.940	0.954	-0.051	0.061	-0.190
MM(ρ_o)	-0.009	-0.012	0.499	0.002	0.944	0.951	-0.014	0.047	-0.587

Scenario 5: $n_s = 10,\ n_t = n_c = 20,\ \rho_w = 0.9,\ \rho_b = 0.$									
UM	-0.147	-0.025	5.717	0.050	0.967	0.989	-0.734	0.089	NA
MM(0)	-0.147	-0.025	5.717	0.052	0.979	0.992	0.219	0.333	0.848
MM(ρ_e)	-0.154	-0.026	5.609	0.051	0.970	0.990	-0.408	0.223	0.516
MM(ρ_o)	-0.157	-0.026	5.581	0.050	0.969	0.990	-0.100	0.218	0.193
Scenario 6: $n_s = 10,\ n_t = n_c \sim U(20,\ 200),\ \rho_w = 0.9,\ \rho_b = 0.$									
UM	-0.049	-0.015	2.720	0.012	0.953	0.976	-0.367	0.053	NA
MM(0)	-0.048	-0.015	2.757	0.012	0.956	0.983	-0.252	0.134	0.685
MM(ρ_e)	-0.048	-0.015	2.698	0.012	0.956	0.983	-0.306	0.113	0.451
MM(ρ_o)	-0.046	-0.015	2.632	0.012	0.961	0.983	-0.170	0.106	0.065
Scenario 7: $n_s = 10,\ n_t = n_c = 20,\ \rho_w = 0.9,\ \rho_b = 0.9.$									
UM	-0.147	-0.026	5.717	0.050	0.967	0.988	-0.734	0.090	NA
MM(0)	-0.147	-0.025	5.717	0.052	0.978	0.991	0.242	0.337	-0.046
MM(ρ_e)	-0.151	-0.026	5.602	0.051	0.969	0.990	-0.420	0.221	-0.380
MM(ρ_o)	-0.153	-0.026	5.577	0.050	0.968	0.989	-0.128	0.216	-0.685
Scenario 8: $n_s = 10,\ n_t = n_c \sim U(20,\ 200),\ \rho_w = 0.9,\ \rho_b = 0.9.$									
UM	-0.049	-0.016	2.720	0.012	0.953	0.981	-0.367	0.053	NA
MM(0)	-0.048	-0.015	2.758	0.012	0.957	0.985	-0.245	0.139	-0.147
MM(ρ_e)	-0.047	-0.015	2.699	0.012	0.956	0.983	-0.308	0.120	-0.384
MM(ρ_o)	-0.045	-0.016	2.636	0.012	0.959	0.983	-0.176	0.106	-0.724
Scenario 9: $n_s = 50,\ n_t = n_c = 200,\ \rho_w = \rho_b = 0.$									
UM	0.009	-0.010	0.450	0.001	0.949	0.952	-0.016	0.021	NA
MM(0)	0.009	-0.010	0.450	0.001	0.949	0.952	-0.016	0.0348	0.019
MM(ρ_e)	0.009	-0.010	0.450	0.001	0.949	0.952	-0.016	0.0349	-0.024
MM(ρ_o)	0.009	-0.010	0.450	0.001	0.949	0.952	-0.016	0.0349	0.015
Scenario 10: $n_s = 50,\ n_t = n_c = 200,\ \rho_w = 0,\ \rho_b = 0.9.$									

UM	0.009	-0.011	0.450	0.001	0.949	0.948	-0.016	0.021	NA
MM(0)	0.009	-0.011	0.450	0.001	0.949	0.951	-0.016	0.036	-0.639
MM(ρ_e)	0.009	-0.011	0.450	0.001	0.949	0.951	-0.016	0.035	-0.751
MM(ρ_o)	0.009	-0.011	0.445	0.001	0.949	0.951	-0.016	0.036	-0.660
Scenario 11: $n_s = 10,\ n_t = n_c = 20,\ \rho_w = \rho_b = 0.$									
UM	-0.147	-0.022	5.717	0.051	0.967	0.99	-0.734	0.094	NA
MM(0)	-0.147	-0.022	5.717	0.051	0.971	0.991	-0.318	0.216	-0.039
MM(ρ_e)	-0.147	-0.022	5.717	0.051	0.970	0.991	-0.316	0.216	-0.056
MM(ρ_o)	-0.147	-0.022	5.717	0.051	0.971	0.991	-0.318	0.216	-0.040
Scenario 12: $n_s = 10,\ n_t = n_c = 20,\ \rho_w = 0,\ \rho_b = 0.9.$									
UM	-0.147	-0.023	5.717	0.051	0.967	0.989	-0.734	0.095	NA
MM(0)	-0.147	-0.022	5.717	0.052	0.972	0.991	-0.319	0.216	-0.917
MM(ρ_e)	-0.147	-0.022	5.717	0.052	0.972	0.991	-0.318	0.216	-0.950
MM(ρ_o)	-0.147	-0.022	5.717	0.0519	0.972	0.991	-0.320	0.216	-0.938

UM: multiple univariate meta-analyses; MM(0): multivariate meta-analyses; MM0-assuming zero as within-study correlations; MM(ρ_e)-assuming common non-zero within-study correlations between treatment effects; MM(ρ_o)-assuming common non-zero within-study correlations between outcomes.

n_t and n_c number of participants in treatment group and control group, respectively; n_s number of studies; τ_1 and τ_2 between-study standard deviation for treatment group and control group, respectively; ρ_b between-study correlation coefficients for overall effects; ρ_w within-study correlation coefficients for outcomes (before dichotomized); p_t and p_c event rates in treatment and control group, respectively.

Supplementary Table B3. Simulation results over 1000 Monte Carlo replications.

True values for between-study parameters $\beta_1 = -2.58$, $\beta_2 = 0.06$, $\tau_1^2 = 4.2^2$, $\tau_2^2 = 0.1^2$. Within-study parameters $\sigma_1^2 = 20^2$, $\sigma_2^2 = 0.5$, $p_t = 0.197$(probability in treatment group), $p_c = 0.192$(probability in control group).

Methods	Bias-M $\hat{\beta}_1$	Bias-M $\hat{\beta}_2$	MSE $\hat{\beta}_1$	MSE $\hat{\beta}_2$	t coverage $\hat{\beta}_1$	t coverage $\hat{\beta}_2$	Bias-M $\hat{\tau}_1$	Bias-M $\hat{\tau}_2$	Bias-M $\hat{\rho}_{12}$
Scenario 13: $n_s = 50,\ n_t = n_c = 200,\ \rho_w = 0.9,\ \rho_b = 0.$									
UM	0.009	-0.012	0.450	0.001	0.949	0.933	-0.016	-0.030	NA
MM(0)	0.009	-0.012	0.450	0.001	0.949	0.940	-0.015	-0.007	0.783
MM(ρ_e)	0.007	-0.012	0.450	0.001	0.949	0.935	-0.015	-0.017	0.482
MM(ρ_o)	0.004	-0.012	0.450	0.001	0.949	0.933	-0.007	-0.020	-0.068
Scenario 14: $n_s = 50,\ n_t = n_c \sim U(20,\ 200),\ \rho_w = 0.9,\ \rho_b = 0.$									
UM	-0.009	-0.015	0.504	0.002	0.944	0.944	-0.046	-0.033	NA
MM(0)	-0.009	-0.015	0.509	0.002	0.946	0.952	-0.014	0.025	0.915
MM(ρ_e)	-0.012	-0.015	0.499	0.002	0.943	0.944	-0.056	-0.011	0.489
MM(ρ_o)	-0.014	-0.015	0.498	0.002	0.943	0.946	-0.022	-0.016	0.017
Scenario 15: $n_s = 50,\ n_t = n_c = 200,\ \rho_w = 0.9,\ \rho_b = 0.9.$									
UM	0.009	-0.012	0.450	0.001	0.949	0.934	-0.016	-0.030	NA
MM(0)	0.009	-0.010	0.450	0.001	0.949	0.955	-0.005	0.038	0.094
MM(ρ_e)	0.010	-0.010	0.450	0.001	0.948	0.950	-0.012	0.003	0.009
MM(ρ_o)	0.010	-0.010	0.450	0.001	0.948	0.946	-0.006	-0.006	-0.067
Scenario 16: $n_s = 50,\ n_t = n_c \sim U(20,\ 200),\ \rho_w = 0.9,\ \rho_b = 0.9.$									
UM	-0.009	-0.015	0.504	0.002	0.944	0.946	-0.046	-0.031	NA
MM(0)	-0.009	-0.013	0.510	0.002	0.948	0.968	0.022	0.074	0.098
MM(ρ_e)	-0.007	-0.013	0.499	0.002	0.940	0.952	-0.041	0.008	-0.051
MM(ρ_o)	-0.007	-0.013	0.499	0.002	0.943	0.949	-0.028	0.001	-0.111

Scenario 17: $n_s = 10,\ n_t = n_c = 20,\ \rho_w = 0.9,\ \rho_b = 0.$									
UM	-0.147	-0.025	5.717	0.051	0.967	0.988	-0.734	0.012	NA
MM(0)	-0.147	-0.025	5.717	0.053	0.979	0.991	0.212	0.255	0.847
MM(ρ_e)	-0.157	-0.026	5.610	0.052	0.970	0.989	-0.412	0.145	0.514
MM(ρ_o)	-0.163	-0.027	5.578	0.051	0.969	0.987	-0.106	0.139	0.185
Scenario 18: $n_s = 10,\ n_t = n_c \sim U(20,\ 200),\ \rho_w = 0.9,\ \rho_b = 0.$									
UM	-0.049	-0.018	2.720	0.013	0.953	0.973	-0.367	-0.015	NA
MM(0)	-0.048	-0.017	2.753	0.013	0.955	0.980	-0.252	0.061	0.657
MM(ρ_e)	-0.049	-0.018	2.692	0.013	0.956	0.979	-0.301	0.041	0.375
MM(ρ_o)	-0.049	-0.018	2.639	0.013	0.959	0.978	-0.173	0.037	0.009
Scenario 19: $n_s = 10,\ n_t = n_c = 20,\ \rho_w = 0.9,\ \rho_b = 0.9.$									
UM	-0.147	-0.027	5.717	0.051	0.967	0.984	-0.734	0.015	NA
MM(0)	-0.147	-0.025	5.717	0.052	0.979	0.990	0.318	0.277	-0.034
MM(ρ_e)	-0.147	-0.025	5.583	0.051	0.969	0.986	-0.443	0.152	-0.321
MM(ρ_o)	-0.144	-0.025	5.559	0.051	0.969	0.988	-0.218	0.142	-0.570
Scenario 20: $n_s = 10,\ n_t = n_c \sim U(20,\ 200),\ \rho_w = 0.9,\ \rho_b = 0.9.$									
UM	-0.049	-0.017	2.720	0.012	0.953	0.981	-0.367	-0.015	NA
MM(0)	-0.048	-0.016	2.762	0.013	0.957	0.988	-0.210	0.089	-0.026
MM(ρ_e)	-0.047	-0.016	2.691	0.012	0.956	0.985	-0.305	0.054	-0.231
MM(ρ_o)	-0.044	-0.016	2.655	0.012	0.959	0.983	-0.211	0.042	-0.448
Scenario 21: $n_s = 50,\ n_t = n_c = 200,\ \rho_w = \rho_b = 0.$									
UM	0.009	-0.013	0.450	0.001	0.949	0.945	-0.016	-0.029	NA
MM(0)	0.009	-0.013	0.450	0.001	0.949	0.946	-0.016	-0.021	0.040
MM(ρ_e)	0.009	-0.013	0.450	0.001	0.949	0.945	-0.016	-0.021	-0.050
MM(ρ_o)	0.009	-0.013	0.450	0.001	0.949	0.946	-0.016	-0.021	0.037
Scenario 22: $n_s = 50,\ n_t = n_c = 200,\ \rho_w = 0,\ \rho_b = 0.9.$									

UM	0.009	-0.013	0.450	0.001	0.949	0.935	-0.016	-0.028	NA
MM(0)	0.009	-0.012	0.450	0.001	0.949	0.944	-0.015	-0.004	-0.107
MM(ρ_e)	0.008	-0.012	0.450	0.001	0.949	0.940	-0.016	-0.014	-0.324
MM(ρ_o)	0.009	-0.012	0.450	0.001	0.949	0.944	-0.015	-0.005	-0.118
Scenario 23: $n_s = 10,\ n_t = n_c = 20,\ \rho_w = \rho_b = 0.$									
UM	-0.147	-0.022	5.717	0.051	0.967	0.990	-0.734	0.021	NA
MM(0)	-0.147	-0.021	5.717	0.052	0.970	0.992	-0.319	0.141	-0.052
MM(ρ_e)	-0.146	-0.021	5.715	0.052	0.969	0.992	-0.310	0.140	-0.007
MM(ρ_o)	-0.147	-0.021	5.717	0.052	0.970	0.992	-0.317	0.141	-0.055
Scenario 24: $n_s = 10,\ n_t = n_c = 20,\ \rho_w = 0,\ \rho_b = 0.9.$									
UM	-0.147	-0.024	5.717	0.051	0.967	0.991	-0.734	0.020	NA
MM(0)	-0.147	-0.023	5.717	0.052	0.972	0.992	-0.304	0.142	-0.826
MM(ρ_e)	-0.148	-0.024	5.717	0.052	0.972	0.992	-0.311	0.142	-0.872
MM(ρ_o)	-0.147	-0.024	5.717	0.052	0.972	0.992	-0.309	0.143	-0.851

UM: multiple univariate meta-analyses; MM(0): multivariate meta-analyses; MM0-assuming zero as within-study correlations; MM(ρ_e)-assuming common non-zero within-study correlations between treatment effects; MM(ρ_o)-assuming common non-zero within-study correlations between outcomes.

n_t and n_c number of participants in treatment group and control group, respectively; n_s number of studies; τ_1 and τ_2 between-study standard deviation for treatment group and control group, respectively; ρ_b between-study correlation coefficients for overall effects; ρ_w within-study correlation coefficients for outcomes (before dichotomized); p_t and p_c event rates in treatment and control group, respectively.

Supplementary Table B4. Simulation results over 1000 Monte Carlo replications.

True values for between-study parameters $\beta_1 = -2.58$, $\beta_2 = 0.06$, $\tau_1^2 = 4.2^2$, $\tau_2^2 = 0.2^2$. Within-study parameters $\sigma_1^2 = 20^2$, $\sigma_2^2 = 0.5$, $p_t = 0.197$(probability in treatment group), $p_c = 0.192$(probability in control group).

Methods	Bias-M $\hat{\beta}_1$	Bias-M $\hat{\beta}_2$	MSE $\hat{\beta}_1$	MSE $\hat{\beta}_2$	t coverage $\hat{\beta}_1$	t coverage $\hat{\beta}_2$	Bias-M $\hat{\tau}_1$	Bias-M $\hat{\tau}_2$	Bias-M $\hat{\rho}_{12}$
Scenario 25: $n_s = 50,\ n_t = n_c = 200,\ \rho_w = 0.9,\ \rho_b = 0.$									
UM	0.009	-0.018	0.450	0.002	0.949	0.925	-0.016	-0.044	NA
MM(0)	0.009	-0.019	0.450	0.002	0.949	0.926	-0.016	-0.042	0.461
MM(ρ_e)	0.003	-0.019	0.450	0.002	0.949	0.926	-0.017	-0.043	0.240
MM(ρ_o)	-0.005	-0.018	0.450	0.002	0.948	0.926	-0.016	-0.043	-0.062
Scenario 26: $n_s = 50,\ n_t = n_c \sim U(20,\ 200),\ \rho_w = 0.9,\ \rho_b = 0.$									
UM	-0.009	-0.022	0.504	0.003	0.944	0.931	-0.046	-0.062	NA
MM(0)	-0.010	-0.022	0.509	0.003	0.945	0.939	-0.019	-0.036	0.745
MM(ρ_e)	-0.018	-0.022	0.500	0.003	0.941	0.929	-0.061	-0.054	0.322
MM(ρ_o)	-0.023	-0.022	0.498	0.003	0.942	0.928	-0.041	-0.055	-0.080
Scenario 27: $n_s = 50,\ n_t = n_c = 200,\ \rho_w = 0.9,\ \rho_b = 0.9.$									
UM	0.009	-0.019	0.450	0.002	0.9490	0.930	-0.016	-0.043	NA
MM(0)	0.009	-0.015	0.450	0.002	0.9490	0.956	0.012	0.006	0.099
MM(ρ_e)	0.007	-0.016	0.449	0.002	0.9480	0.939	-0.018	-0.035	-0.004
MM(ρ_o)	0.008	-0.016	0.449	0.002	0.9480	0.939	-0.017	-0.034	0.002
Scenario 28: $n_s = 50,\ n_t = n_c \sim U(20,\ 200),\ \rho_w = 0.9,\ \rho_b = 0.9.$									
UM	-0.009	-0.022	0.504	0.003	0.944	0.932	-0.046	-0.058	NA
MM(0)	-0.010	-0.017	0.510	0.003	0.949	0.958	0.063	0.036	0.100
MM(ρ_e)	-0.008	-0.018	0.500	0.003	0.943	0.943	-0.038	-0.039	-0.026
MM(ρ_o)	-0.008	-0.018	0.501	0.003	0.941	0.943	-0.045	-0.033	0.011

Scenario 29: $n_s = 10,\ n_t = n_c = 20,\ \rho_w = 0.9,\ \rho_b = 0.$									
UM	-0.149	-0.032	5.717	0.053	0.967	0.985	-0.73	-0.065	NA
MM(0)	-0.147	-0.031	5.717	0.055	0.977	0.989	0.221	0.168	0.846
MM(ρ_e)	-0.168	-0.033	5.612	0.053	0.97	0.985	-0.414	0.062	0.511
MM(ρ_o)	-0.179	-0.033	5.575	0.053	0.969	0.983	-0.120	0.055	0.138
Scenario 30: $n_s = 10,\ n_t = n_c \sim U(20,\ 200),\ \rho_w = 0.9,\ \rho_b = 0.$									
UM	-0.049	-0.025	2.720	0.016	0.953	0.956	-0.367	-0.076	NA
MM(0)	-0.048	-0.024	2.750	0.016	0.955	0.968	-0.250	-0.013	0.565
MM(ρ_e)	-0.053	-0.025	2.690	0.016	0.956	0.965	-0.296	-0.029	0.287
MM(ρ_o)	-0.058	-0.025	2.645	0.016	0.959	0.96	-0.193	-0.030	-0.040
Scenario 31: $n_s = 10,\ n_t = n_c = 20,\ \rho_w = 0.9,\ \rho_b = 0.9.$									
UM	-0.147	-0.036	5.717	0.053	0.967	0.982	-0.73	-0.071	NA
MM(0)	-0.147	-0.031	5.717	0.055	0.979	0.990	0.438	0.209	-0.006
MM(ρ_e)	-0.147	-0.031	5.564	0.053	0.970	0.986	-0.482	0.071	-0.266
MM(ρ_o)	-0.141	-0.030	5.538	0.053	0.970	0.986	-0.327	0.054	-0.470
Scenario 32: $n_s = 10,\ n_t = n_c \sim U(20,\ 200),\ \rho_w = 0.9,\ \rho_b = 0.9.$									
UM	-0.049	-0.024	2.720	0.015	0.953	0.967	-0.367	-0.073	NA
MM(0)	-0.050	-0.020	2.758	0.015	0.959	0.983	-0.144	0.040	0.037
MM(ρ_e)	-0.046	-0.020	2.683	0.015	0.956	0.975	-0.297	-0.011	-0.176
MM(ρ_o)	-0.044	-0.020	2.672	0.015	0.956	0.974	-0.259	-0.016	-0.233
Scenario 33: $n_s = 50,\ n_t = n_c = 200,\ \rho_w = \rho_b = 0.$									
UM	0.009	-0.020	0.450	0.002	0.949	0.915	-0.016	-0.043	NA
MM(0)	0.009	-0.020	0.450	0.002	0.949	0.916	-0.016	-0.042	0.010
MM(ρ_e)	0.008	-0.020	0.450	0.002	0.949	0.915	-0.016	-0.042	-0.060
MM(ρ_o)	0.009	-0.020	0.450	0.002	0.949	0.916	-0.016	-0.042	0.009
Scenario 34: $n_s = 50,\ n_t = n_c = 200,\ \rho_w = 0,\ \rho_b = 0.9.$									

UM	0.009	-0.019	0.450	0.002	0.949	0.917	-0.016	-0.041	NA
MM(0)	0.009	-0.017	0.4450	0.002	0.949	0.934	-0.013	-0.031	-0.016
MM(ρ_e)	0.005	-0.018	0.449	0.002	0.949	0.928	-0.018	-0.039	-0.278
MM(ρ_o)	0.009	-0.017	0.450	0.002	0.949	0.934	-0.014	-0.031	-0.022
Scenario 35: $n_s = 10,\ n_t = n_c = 20,\ \rho_w = \rho_b = 0.$									
UM	-0.147	-0.030	5.717	0.052	0.967	0.991	-0.734	-0.066	NA
MM(0)	-0.147	-0.029	5.717	0.053	0.970	0.992	-0.297	0.054	-0.046
MM(ρ_e)	-0.144	-0.029	5.713	0.053	0.971	0.992	-0.285	0.054	0.049
MM(ρ_o)	-0.147	-0.029	5.717	0.053	0.970	0.992	-0.298	0.054	-0.046
Scenario 36: $n_s = 10,\ n_t = n_c = 20,\ \rho_w = 0,\ \rho_b = 0.9.$									
UM	-0.147	-0.035	5.717	0.053	0.967	0.988	-0.734	-0.067	NA
MM(0)	-0.147	-0.034	5.717	0.054	0.972	0.991	-0.280	0.056	-0.730
MM(ρ_e)	-0.149	-0.034	5.718	0.054	0.972	0.991	-0.292	0.056	-0.810
MM(ρ_o)	-0.147	-0.034	5.717	0.054	0.973	0.991	-0.285	0.056	-0.752

UM: multiple univariate meta-analyses; MM(0): multivariate meta-analyses; MM0-assuming zero as within-study correlations; MM(ρ_e)-assuming common non-zero within-study correlations between treatment effects; MM(ρ_o)-assuming common non-zero within-study correlations between outcomes.

n_t and n_c number of participants in treatment group and control group, respectively; n_s number of studies; τ_1 and τ_2 between-study standard deviation for treatment group and control group, respectively; ρ_b between-study correlation coefficients for overall effects; ρ_w within-study correlation coefficients for outcomes (before dichotomized); p_t and p_c event rates in treatment and control group, respectively.

Supplementary Figure B1 Approximation errors for continuous outcomes (mean squared error): formulae 1.2 (left) and 1.6 (right). Each line represents a simulation scenario according to variable number of participants $n_t = n_c = 20$ (purple), 50 (green), 100 (blue), 200 (red), 1000 (black).

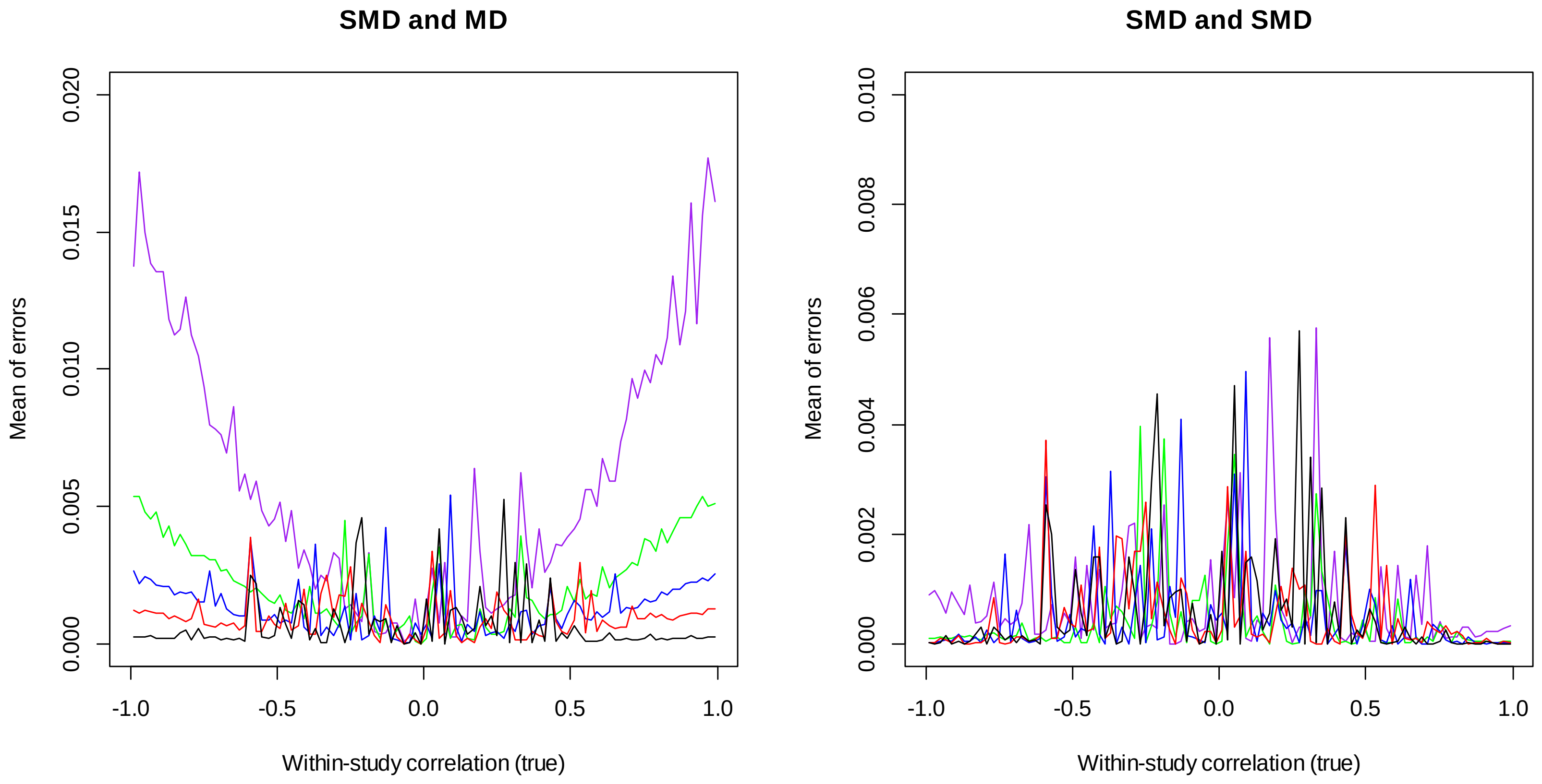

Supplementary Figure B2 Approximation errors for continuous and dichotomous outcomes (mean squared error): formulae 1.3 (top left), 1.4 (top middle), 1.5 (top right); and formulae 1.7 (bottom left), 1.8 (bottom middle) and 1.9 (bottom right), with sample size $n_t = n_c = 20$ (purple), 50 (green), 100 (blue), 200 (red), 1000 (black).

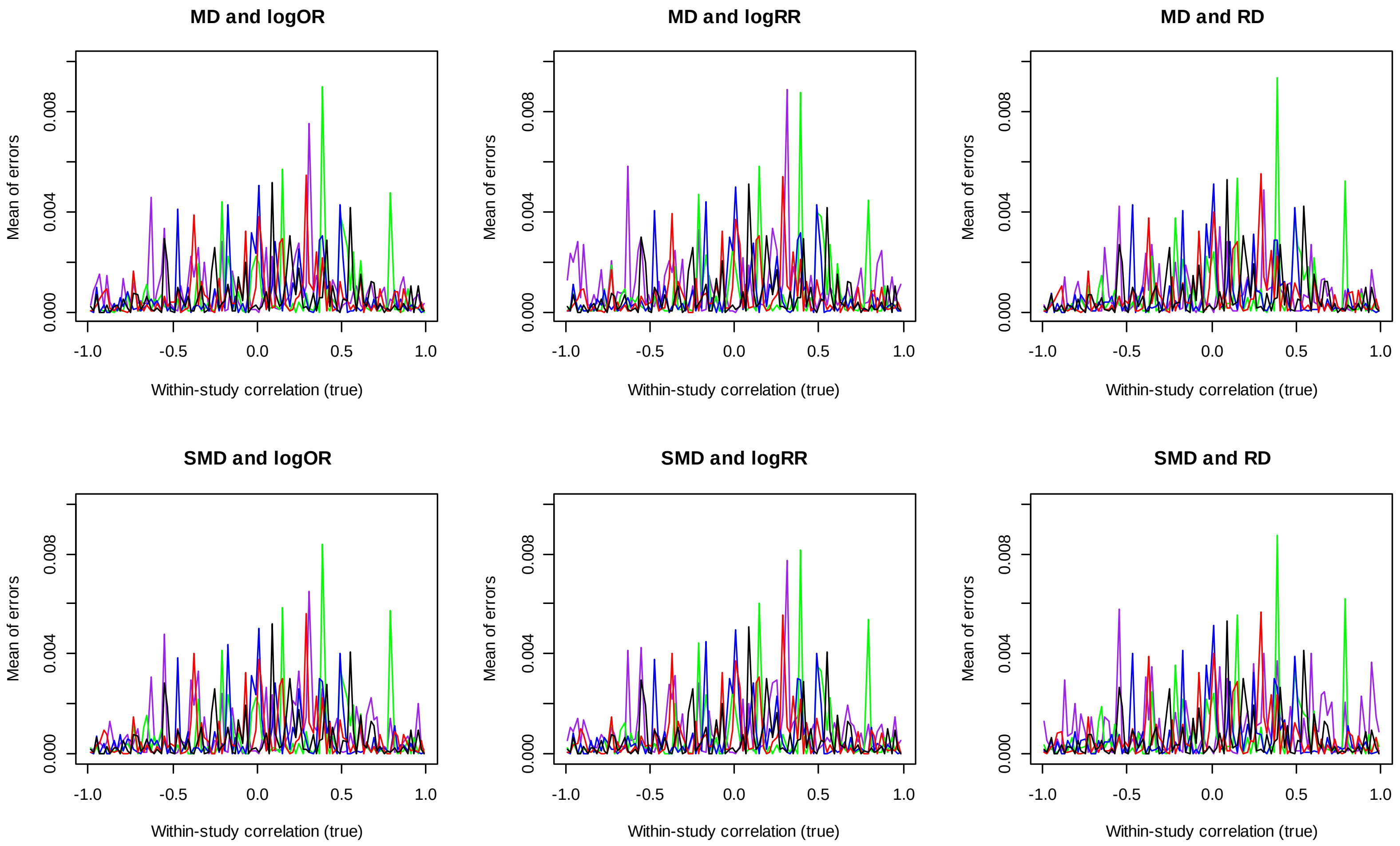

Supplementary Figure B3 Approximation errors for dichotomous outcomes (mean squared error): formulae 1.10 (top left), 1.11 (top middle), 1.12 (top right); and formula 1.13 (bottom left), 1.14 (bottom middle) and 1.15 (bottom right), with sample size $n_t = n_c = 20$ (purple), 50 (green), 100 (blue), 200 (red), 1000 (black).

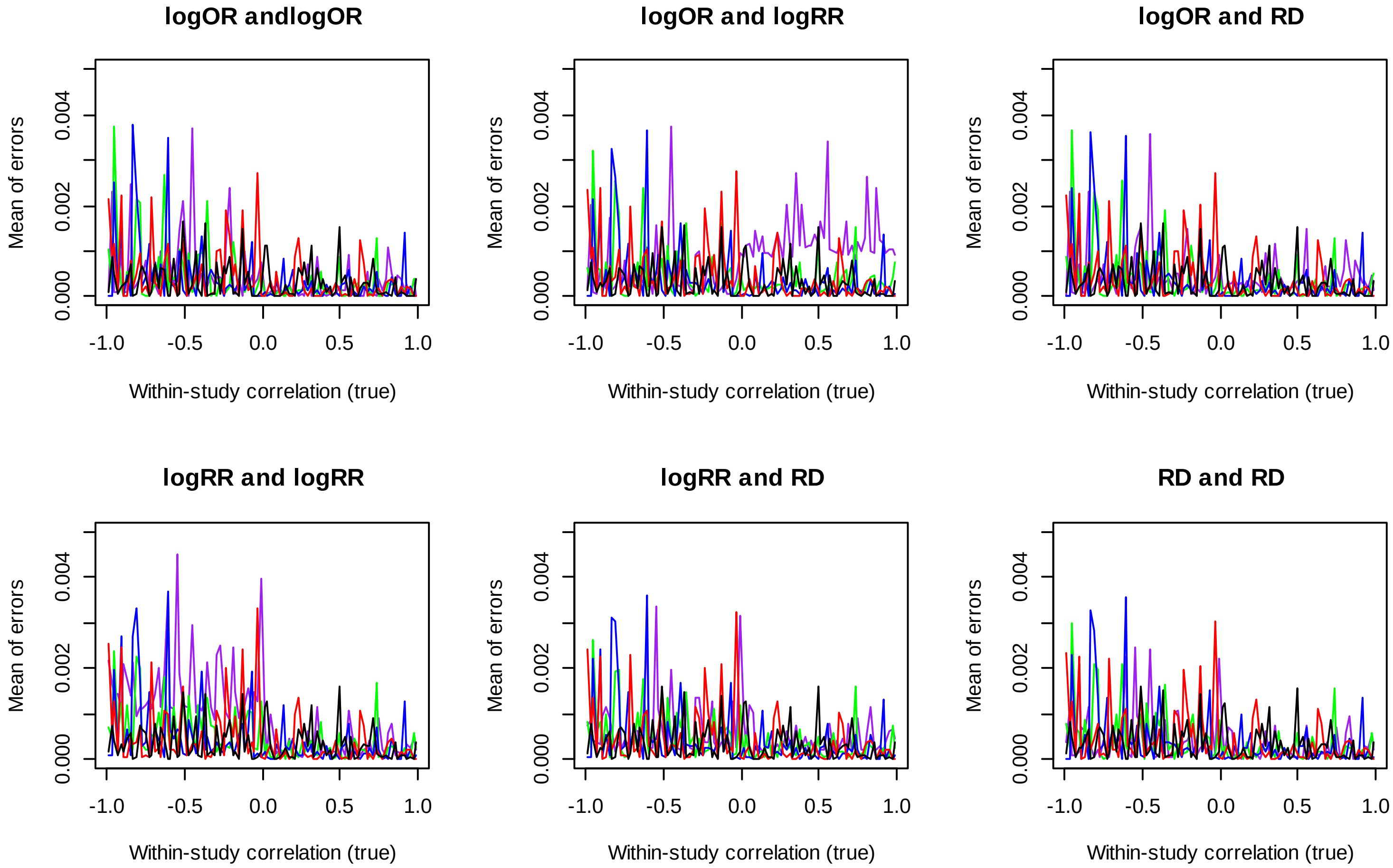

SUPPLEMENTARY MATERIAL C

Supplementary Table C1 Data for SBP and DBP in trials of vasoactive drugs for acute stroke [4].

			SBP						DBP					
Study	N_t	N_c	Treatment			Control			Treatment			Control		
			N	Mean	SD	N	Mean	SD	N	Mean	SD	N	Mean	SD
Barer 1988 atenolol	18	11	18	138.06	24.68	10	140.53	23.27	18	77.61	11.34	10	81.05	14.39
Barer 1988 propanolol	16	11	14	142.14	30.93	9	140.53	23.27	14	80.71	16.51	9	81.05	14.39
Barer 1988/50 mg	102	50	95	137.31	25.14	47	145.47	22.58	95	79.63	13.62	47	86.07	15.23
Barer 1988/80 mg	101	50	97	142.3	23.85	48	145.47	22.58	97	82.66	13.93	48	86.07	15.23
ASCLEPIOS 1990	120	114	107	144.73	23.77	97	144.88	23.96	107	80.98	11.83	97	83.37	13.5
Limburg 1990	12	14	10	141	26.65	6	150.83	24.58	10	86.1	18.53	6	84.17	13.57
Norris 1994	96	93	83	133.62	16.3	75	149.87	25.1	83	71.41	10.33	75	83.29	12.42
Bogousslavsky 1990	24	28	24	134	24	28	141	16	24	81	11	28	80	11
Kaste 1994/120 mg	176	174	160	146.6	25.81	163	148.5	25.81	160	84.3	14.84	161	89.2	14.24
Lowe 1993	56	56	54	145.19	24.65	54	141.02	22.39	54	85.11	13.29	54	81.3	11.33
Paci 1989/120 mg	19	22	19	136.7	17.4	22	145.7	19.7	19	82.8	10	22	85.2	9.8
Squire 1996	75	72	68	145.72	22.65	55	147.85	21.82	68	81.35	12.66	55	82.53	11.26
VENUS 1995	225	229	215	152.69	25.31	213	152.16	26.5	215	84.16	12.81	213	85.81	13.54
Lees 1995	30	30	27	147.48	28.27	25	139.52	23.82	27	79.63	17.24	25	76.92	15.79
IMAGES Pilot	26	25	26	147.35	17.54	25	155.94	27.31	26	72.94	10.12	25	82.12	10.9
Muir 1995	19	6	15	150.93	20.33	4	159.25	22.02	15	82.2	15.49	4	89.75	15.11
Strand 1984	13	13	13	168.46	35.73	12	150.42	18.4	13	84.62	15.87	12	80.83	10.19
PRISTINE	313	307	310	155.7	24.45	307	154.33	24.95	310	87.8	12.55	307	85.96	12.64
Steiner 1986	55	45	44	139.89	20.01	37	147.3	26.21	44	82.73	10.37	37	86.89	15.56
Herrschaft 1988	24	20	23	176.6	19.6	17	174.2	20.2	23	94.5	12.4	17	95.2	10.2
Huczynski 1988	15	15	15	140.4	27.84	15	146.33	26.94	15	87	11.19	15	86.47	17.31

Supplementary Table C2 Data for outcomes of death and ‘death and disability’ in trials of vasoactive drugs for acute stroke [4].

			Death				Death or disability			
Study	N_t	N_c	Treatment		Control		Treatment		Control	
			N	n	N	n	N	n	N	n
Barer 1988 atenolol	18	11	18	4	11	4	16	8	10	5
Barer 1988 propanolol	16	11	16	7	10	3	16	11	10	5
Barer 1988/50 mg	102	50	102	37	50	12	101	45	50	21
Barer 1988/80 mg	101	50	100	33	50	12	100	48	50	22
ASCLEPIOS 1990	120	114	116	21	114	19	116	47	114	44
Limburg 1990	12	14	12	3	14	5	12	3	14	7
Norris 1994	96	93	96	29	93	33	90	39	79	42
Bogousslavsky 1990	24	28	24	0	28	1	24	6	28	4
Kaste 1994/120 mg	176	174	176	29	174	22	175	44	172	31
Lowe 1993	56	56	56	15	56	12	40	25	46	18
Paci 1989/120 mg	19	22	19	0	22	0	19	1	22	4
Squire 1996	75	72	75	12	72	17	69	32	63	32
VENUS 1995	225	229	225	30	229	32	223	63	225	57
Lees 1995	30	30	30	6	30	7	30	9	30	12
IMAGES Pilot	26	25	26	3	25	6	26	12	25	11
Muir 1995	19	6	19	1	6	0	19	3	6	1
Strand 1984	13	13	13	2	13	3	13	6	13	9
PRISTINE	313	307	307	49	303	41	307	184	303	182
Steiner 1986	55	45	55	21	45	16	55	38	45	33
Herrschaft 1988	24	20	23	0	17	0	23	8	17	10
Huczynski 1988	15	15	15	4	15	1	14	4	15	2

Supplementary Table C3 Effect size and approximate within-study covariance matrices for bivariate meta-analyses of acute stroke data [4]

Study	Bivariate meta-analysis of SBP and DBP					Bivariate meta-analysis of death (D) and 'death or disability' (DD)				
	Mean difference in SBP	Mean difference in DBP	Var(MD_{SBP})	Var(MD_{DBP})	Cov(MD_{SBP}, MD_{DBP})	Log OR for D	Log OR for DD	Var($logOR_D$)	Var($logOR_{DD}$)	Cov($logOR_D$, $logOR_{DD}$)
Barer 1988 atenolol	-2.47	-3.44	87.99	27.85	34.81	-0.693	0	0.714	0.650	0.504
Barer 1988 propanolol	1.61	-0.34	128.50	42.48	52.31	0.596	0.788	0.730	0.691	0.447
Barer 1988/50 mg	-8.16	-6.44	17.50	6.89	7.75	0.589	0.104	0.152	0.122	0.098
Barer 1988/80 mg	-3.17	-3.41	16.49	6.83	7.52	0.444	0.161	0.155	0.121	0.091
ASCLEPIOS 1990	-0.15	-2.39	11.20	3.187	4.23	0.100	0.080	0.121	0.073	0.053
Limburg 1990	-9.83	1.93	171.72	65.03	74.53	-0.511	-1.099	0.756	0.730	0.667
Norris 1994	-16.25	-11.88	11.60	3.34	4.39	-0.24	-0.395	0.096	0.096	0.079
Bogousslavsky 1990	-7	1	33.14	9.36	12.27	-0.554	0.693	3.09-	0.514	0.426
Kaste 1994/120 mg	-1.9	-4.9	8.25	2.64	3.30	0.310	0.424	0.093	0.070	0.064
Lowe 1993	4.17	3.81	20.54	5.65	7.64	0.294	0.953	0.197	0.198	0.14
Paci 1989/120 mg	-9	-2.4	33.58	9.63	12.73	0.150	-1.386	4.101	1.361	1.25
Squire 1996	-2.13	-1.18	16.20	4.66	6.17	-0.484	-0.177	0.176	0.122	0.082
VENUS 1995	0.53	-1.65	6.28	1.62	2.27	-0.054	0.149	0.075	0.046	0.039
Lees 1995	7.96	2.71	52.30	20.98	23.50	-0.197	-0.442	0.395	0.298	0.248
IMAGES Pilot	-8.59	-9.18	41.67	8.69	13.30	-0.884	0.087	0.596	0.317	0.214
Muir 1995	-8.32	-7.55	148.77	73.07	73.96	-0.492	-0.065	3.237	1.596	1.352
Strand 1984	18.04	3.79	126.42	28.03	42.06	-0.501	-0.965	1.024	0.671	0.341
PRISTINE	1.37	1.84	3.96	1.03	1.43	0.194	-0.005	0.053	0.027	0.013
Steiner 1986	-7.41	-4.16	27.67	8.99	11.17	0.113	-0.207	0.174	0.199	0.09
Herrschaft 1988	2.4	-0.7	40.71	12.81	16.11	-0.310	-0.985	4.105	0.435	0.225
Huczynski 1988	-5.93	0.53	100.06	28.32	36.82	1.627	0.956	1.412	0.927	0.886

SBP = systolic blood pressure, DBP = diastolic blood pressure, D = death, DD = death or disability, MD = mean difference OR = odds ratio
