# Supplementary material for: Estimating within-study covariances in multivariate meta-analysis with multiple outcomes
Source: Stat Med. 2012 Dec 3;32(7):1191–205. doi: 10.1002/sim.5679 (PMC3618374; doi:10.1002/sim.5679)
Supplement: Supplementary file 2 [file sim0032-1191-SD2.doc]

# Supplementary materials for “Estimating within-study covariances in multivariate meta-analysis with multiple outcomes”

YINGHUI WEI[[1]](#footnote-2), JULIAN PT HIGGINS

**Supplementary material A**

Derivation of covariances between particular pairs of summary statistics

### A1. Derivation of equation (6)

We first derive equation (6), the covariance between two sample probabilities for two outcomes within a treatment group. Let $I_{c1i},i=1,2,...,n_{1c}$ and $I_{c2i},i=1,2,...,n_{2c}$ denote Bernoulli variables, which take value 1 for the event of interest with probabilities $p_{1c}$ and $p_{2c}$ and value 0 otherwise. Given knowledge of the correlation between the dichotomous variables, i.e. $\rho=corr(I_{c1i},I_{c2i})$, we can deduce that

$$\begin{matrix} \mathrm{cov}(\hat{p}_{1c},\hat{p}_{2c})=\mathrm{cov}\left(\frac{S_{1c}}{n_{1c}},\frac{S_{2c}}{n_{2c}}\right) \\ =\frac{1}{n_{1c}n_{2c}}\mathrm{cov}(S_{1c},S_{2c}) \\ =\frac{1}{n_{1c}n_{2c}}\mathrm{cov}\left(\sum_{i=1}^{n_{1c}} I_{1ci},\sum_{i=1}^{n_{2c}} I_{2ci}\right) \\ =\frac{1}{n_{1c}n_{2c}}\cdot\sum_{i=i'} \mathrm{cov}(I_{1ci},I_{2ci'}) \\ =\frac{n_{12c}}{n_{1c}n_{2c}}\rho\sqrt{p_{1c}(1-p_{1c})p_{2c}(1-p_{2c})} \end{matrix}$$

and similarly

$\mathrm{cov}(\hat{p}_{1t},\hat{p}_{2t})=\frac{n_{12t}}{n_{1t}n_{2t}}\rho\sqrt{p_{1t}(1-p_{1t})p_{2t}(1-p_{2t})}$.

### A2. Derivation of equation (9)

We now derive equation (9), the covariance term between probabilities for two outcomes, given that outcome 1 is nested within outcome 2. We illustrate the covariance term for the treatment group; the covariance term for the control group can be derived in a similar way.

Suppose we observe $S_{1t}$ out of *N_t_* participants with outcome 1, and $S_{2t}$ out of *N_t_* participants with outcome 2. Since outcome 1 is nested within outcome 2, we have $S_{2t}=S_{1t}+a_{t}$ $(a_{t}\geq0)$ and $p_{2t}=p_{1t}+p_{at}$, with $p_{1t}$, $p_{2t}$ and $p_{at}$ being the probabilities of events $S_{1t}$, $S_{2t}$ and $a_{t}$ respectively. We assume a multinomial distribution: $(S_{1t},a_{t})\sim multinomial(N_{t},p_{1t},p_{at})$. Then

$$\begin{matrix} \mathrm{cov}(p_{1t},p_{2t})=\mathrm{cov}\left(\frac{S_{1t}}{N_{t}},\frac{S_{2t}+a_{t}}{N_{t}}\right) \\ =\mathrm{cov}\left(\frac{S_{1t}}{N_{t}},\frac{S_{1t}+a_{t}}{N_{t}}\right) \\ =\frac{1}{N_{t}^{2}}\mathrm{cov}(S_{1t},S_{1t}+a_{t}) \\ =\frac{1}{N_{t}^{2}}\mathrm{var}(S_{1t})+\frac{1}{N_{t}^{2}}\mathrm{cov}(S_{1t},a_{t}) \\ =\frac{1}{N_{t}}p_{1t}(1-p_{1t})-\frac{1}{N_{t}}p_{1t}p_{at} \end{matrix}$$

In practice, we might observe different denominators ($n_{1t},n_{2t}$) for outcomes 1 and 2. Typically this situation will be associated with $n_{2t}<n_{1t}$, since information on event ‘A or B’ should more readily be available than information on event ‘A alone’. Thus we take $N_{t}=n_{1t}$. For the $n_{1t}-n_{2t}$ participants for whom we do not know their outcomes, we assume they are missing at random and assign a proportion $\left(S_{2t}-S_{1t}\right)/\left(n_{2t}-S_{1t}\right)$ of them to event ‘B not A’ (equivalently ‘A or B’) and the remainder to event ‘neither A nor B’. This yields a revised value of *a_t_*, say $a'_{t}=a_{t}+(n_{1t}-n_{2t})\times\frac{S_{2t}-S_{1t}}{n_{2t}-S_{1t}}$.

### A3 Derivation of equation (12)

Now we derive equation (12), the covariance between two inverse sample variances for two continuous outcomes within a study. We use the properties of the bivariate chi-squared distribution to assist this deviation.

#### Bivariate chi-squared distribution

Let $(Z_{1},Z_{2})$ be a two dimensional correlated random vector with mean $(\mu_{1},\mu_{2}){}^{T}$ and variance-covariance matrix $\mathbf{\Sigma}$ having elements $\sigma_{ik}$ for *i* = 1, 2; *k* = 1, 2, where $\sigma_{11}=\sigma_{1}^{2}$, $\sigma_{22}=\sigma_{2}^{2}$, $\sigma_{12}=\rho\sigma_{1}\sigma_{2}$, and $\rho$ is a correlation coefficient. Let $s_{1}^{2}$ and $s_{2}^{2}$ be sample variances for realizations of *Z*_1_ and *Z*_2_. Then random quantities $U=vs_{1}^{2}/\sigma_{1}^{2}$ and $V=vs_{2}^{2}/\sigma_{2}^{2}$ follow a bivariate chi-squared distribution with probability density function described by Joarder (2007; 2008a; 2008b). Two useful properties are:

Property 1: $\text{corr}(U,V)=\frac{\mathrm{cov}(U,V)}{\sqrt{\mathrm{var}(U)\mathrm{var}(V)}}=\rho^{2}$

Property 2: $\mathrm{var}(U)=\mathrm{var}(V)=2v$

Now consider the situation of two outcomes being measured in each of two treatment arms. We use $s_{ja}^{2}$ to denote the sample variance for outcome *j* = 1 or 2 in treatment arm *a* = *t* or *c*. The pooled sample variance can be written as

$$s_{jp}^{2}=\lambda_{jt}\cdot s_{jt}^{2}+\lambda_{jc}\cdot s_{jc}^{2}$$

where $\lambda_{ja}=v_{ja}/\left(n_{jc}+n_{jt}-2\right)$, with $v_{ja}=n_{ja}-1$ being the degrees of freedom for outcome *j* in treatment arm *a*. The variances of the pooled sample variances are

$$\begin{matrix} \mathrm{var}(s_{1p}^{2})=\mathrm{var}(\lambda_{1t}s_{1t}^{2}+\lambda_{1c}s_{1c}^{2}) \\ =\lambda_{1t}^{2}\mathrm{var}(s_{1t}^{2})+\lambda_{1c}^{2}\mathrm{var}(s_{1c}^{2}) \\ =2\lambda_{1t}^{2}\sigma_{1}^{4}v_{1t}^{-1}+2\lambda_{1c}^{2}\sigma_{1}^{4}v_{1c}^{-1} \\ =2(\lambda_{1t}^{2}v_{1t}^{-1}+\lambda_{1c}^{2}v_{1c}^{-1})\sigma_{1}^{4} \\ =2(\lambda_{t}^{2}v_{t}^{-1}+\lambda_{c}^{2}v_{c}^{-1})\sigma_{1}^{4}\text{ (if }n_{1t}=n_{2t}\text{ and }n_{1c}=n_{2c}) \end{matrix}$$

and

$$\mathrm{var}(s_{2p}^{2})=2(\lambda_{t}^{2}v_{t}^{-1}+\lambda_{c}^{2}v_{c}^{-1})\sigma_{2}^{4}\text{ (if }n_{1t}=n_{2t}\text{ and }n_{1c}=n_{2c})$$

To obtain the covariances, we first apply Property 1 to the sample variances:

$$\begin{matrix} corr(s_{1a}^{2},s_{2a}^{2})=\frac{\mathrm{cov}(s_{1a}^{2},s_{2a}^{2})}{\sqrt{\mathrm{var}(s_{1a}^{2})\mathrm{var}(s_{2a}^{2})}} \\ =\frac{\mathrm{cov}(U\sigma_{1}^{2}/v_{1a},U\sigma_{2}^{2}/v_{2a})}{\sqrt{\mathrm{var}(U\sigma_{1}^{2}/v_{1a})\mathrm{var}(U\sigma_{2}^{2}/v_{2a})}} \\ =\frac{\mathrm{cov}(U,V)}{\sqrt{\mathrm{var}(U)\mathrm{var}(V)}} \\ =\rho^{2} \end{matrix}$$

and then derive

$$\begin{matrix} \mathrm{cov}(s_{1p}^{2},s_{2p}^{2})=\mathrm{cov}(\lambda_{1t}s_{1t}^{2}+\lambda_{1c}s_{1c}^{2},\lambda_{2t}s_{2t}^{2}+\lambda_{2c}s_{2c}^{2}) \\ =\lambda_{1t}\lambda_{2t}\mathrm{cov}(s_{1t}^{2},s_{2t}^{2})+\lambda_{1c}\lambda_{2c}\mathrm{cov}(s_{1c}^{2},s_{2c}^{2}) \\ =\lambda_{1t}\lambda_{2t}\cdot2\rho^{2}v_{12t}\cdot\sigma_{1}^{2}v_{12t}^{-1}\sigma_{2}^{2}v_{12t}^{-1}+\lambda_{1c}\lambda_{2c}\cdot2\rho^{2}v_{12c}\cdot\sigma_{1}^{2}v_{12c}^{-1}\sigma_{2}^{2}v_{12c}^{-1} \\ =2\lambda_{1t}\lambda_{2t}v_{12t}^{-1}\rho^{2}\sigma_{1}^{2}\sigma_{2}^{2}+2\lambda_{1c}\lambda_{2c}v_{12c}^{-1}\rho^{2}\sigma_{1}^{2}\sigma_{2}^{2} \\ =2(\lambda_{1t}\lambda_{2t}v_{12t}^{-1}+\lambda_{1c}\lambda_{2c}v_{12c}^{-1})\rho^{2}\sigma_{1}^{2}\sigma_{2}^{2} \end{matrix}$$

Let $k_{1}=2(\lambda_{1t}^{2}v_{1t}^{-1}+\lambda_{1c}^{2}v_{1c}^{-1})$, $k_{2}=2(\lambda_{2t}^{2}v_{2t}^{-1}+\lambda_{2c}^{2}v_{2c}^{-1})$, and $k_{12}=2(\lambda_{1t}\lambda_{2t}v_{12t}^{-1}+\lambda_{1c}\lambda_{2c}v_{12c}^{-1})$. Then the covariance matrix for $s_{1p}^{2}$and $s_{2p}^{2}$ is given by

$\mathbf{\Sigma}^{*}=\left[\begin{matrix} k_{1}\sigma_{1}^{4} & k_{12}\rho^{2}\sigma_{1}^{2}\sigma_{2}^{2} \\ k_{12}\rho^{2}\sigma_{1}^{2}\sigma_{2}^{2} & k_{2}\sigma_{2}^{4} \end{matrix}\right]$.

We derive the desired $\mathrm{cov}\left(s_{1p}^{-1},s_{2p}^{-1}\right)$ using the delta method as described in the main text. Writing $\mathbf{X}=(s_{1p}^{2},s_{2p}^{2})$ we take $f_{1}(\mathbf{X})=s_{1p}^{-1}$ and $f_{2}(\mathbf{X})=s_{2p}^{-1}$, then

$\nabla f^{*}(\mathbf{X})=\left[\begin{matrix} -\frac{1}{2}s_{1p}^{-\frac{3}{2}} & 0 \\ 0 & -\frac{1}{2}s_{1p}^{-\frac{3}{2}} \end{matrix}\right]$,

$$\begin{matrix} \mathbf{\Sigma}^{**}=\nabla f^{*T}\mathbf{\Sigma}^{*}\nabla f^{*} \\ =\left[\begin{matrix} -\frac{1}{2}\sigma_{1}^{-3} & 0 \\ 0 & -\frac{1}{2}\sigma_{2}^{-3} \end{matrix}\right]\mathbf{\Sigma}^{*}\left[\begin{matrix} -\frac{1}{2}\sigma_{1}^{-3} & 0 \\ 0 & -\frac{1}{2}\sigma_{2}^{-3} \end{matrix}\right] \\ =\left[\begin{matrix} \frac{1}{4}k_{1}\sigma_{1}^{-2} & \frac{1}{4}k_{12}\rho^{2}\sigma_{1}^{-1}\sigma_{2}^{-1} \\ \frac{1}{4}k_{12}\rho^{2}\sigma_{1}^{-1}\sigma_{2}^{-1} & \frac{1}{4}k_{2}\sigma_{2}^{-2} \end{matrix}\right] \end{matrix}$$

That is

$corr(s_{1p}^{-1},s_{2p}^{-1})=\frac{k_{12}}{\sqrt{k_{1}k_{2}}}\rho^{2}$.

Therefore, by using the approximate variance for $s_{1p}^{-1}$ and $s_{2p}^{-1}$, we have

$$\begin{matrix} \mathrm{cov}(s_{1p}^{-1},s_{2p}^{-1})=\frac{k_{12}}{\sqrt{k_{1}k_{2}}}\rho^{2}\sqrt{\mathrm{var}(s_{1p}^{-2})\mathrm{var}(s_{2p}^{-2})} \\ \text{ }=\frac{k_{12}}{\sqrt{k_{1}k_{2}}}\rho^{2}\frac{1}{\sigma_{1}\sigma_{2}}\sqrt{\left(\frac{v_{1}}{v_{1}-2}-\frac{1}{J(v_{1})^{2}}\right)\left(\frac{v_{2}}{v_{2}-2}-\frac{1}{J(v_{2})^{2}}\right)}. \end{matrix}$$

### A4. Derivation of equation (13)

Finally we derive equation (13) the covariance between an estimated mean difference and a sample probability.

$$\begin{matrix} \mathrm{cov}\left(\bar{y}_{1c}-\bar{y}_{1t},\hat{p}_{2c}\right)=\mathrm{cov}\left(\bar{y}_{1c},\hat{p}_{2c}\right) \\ =\mathrm{cov}\left(\bar{y}_{1c},\frac{S_{2c}}{n_{2c}}\right) \\ =\mathrm{cov}\left(\frac{1}{n_{1c}}\sum_{i=1}^{n_{1c}} y_{1ci},\frac{1}{n_{2c}}\sum_{i=1}^{n_{2c}} I_{2ci}\right) \\ =\frac{1}{n_{1c}n_{2c}}\sum_{i=i'} \mathrm{cov}\left(y_{1ci},I_{2ci'}\right)\text{ } \\ =\frac{n_{12c}}{n_{1c}n_{2c}}\rho s_{1c}\sqrt{p_{2c}(1-p_{2c})}\text{ } \end{matrix}$$

since $I_{2ci}$ follows a Bernoulli distribution. Similarly,

$\mathrm{cov}\left(\bar{y}_{1c}-\bar{y}_{1t},\hat{p}_{2t}\right)\text{ }=\frac{n_{12t}}{n_{1t}n_{2t}}\rho s_{1t}\sqrt{p_{2t}(1-p_{2t})}\text{ }$.

**References for supplementary material A**

Joarder AH. A bivariate chi-square distribution and some of its properties. KFUPM, Department of Mathematics & Statistics, *Technical report*, 2007.

Joarder AH, Abujiya MR. Standardized moments for bivariate chi-square distribution. *Journal of Applied Statistical Science* 2008a; **16**:1-9.

Joarder AH and Omar MH. Some Statistical Properties of a Bivariate Chi-Square Probability Density Function. KFUPM, Department of Mathematics & Statistics, *Technical report* 382, 2008b.

**Supplementary material B**

### B1 Simulation procedures

*Overview* We simulate meta-analysis data for two outcomes, with outcome 1 being a continuous variable and outcome 2 being a dichotomous variable. The treatment effects are measured using a mean difference and a log odds ratio for the two outcomes respectively. To induce correlation between the outcomes within studies, we simulate individual participant outcomes from a bivariate normal distribution and dichotomize the second variable. The correlations between the continuous and dichotomous outcomes, and between the treatment effect estimates for the two outcomes, are estimated empirically.

*Simulation parameter specification* We consider a wide range of sample sizes to assess the dependence of estimation properties on the number of studies ($N_{s}$), the number of participants in treatment ($N_{t}$) and control groups ($N_{c}$), as well as the degree of dependence between outcomes. We approximately mimic the SBP and DBP outcomes in the acute stroke data by setting the between-study parameters as

$$\theta_{1}=-2.58,\text{ }\theta_{2}=0.06,\text{ }\tau_{1}^{2}=4.20^{2},\text{ }\tau_{2}^{2}=0.000,$$

and within-study (between-participant) variances as

$\sigma_{1}^{2}=20^{2},\text{ }\sigma_{2}^{2}=0.5$.

To further evaluate the impact of the heterogeneity on the parameters estimates, we extend the simulation to consider $\tau_{2}^{2}=0.1^{2}$ and $\tau_{2}^{2}=0.2^{2}$, while keeping the other parameters unchanged. We evaluate combinations of within-study correlation ($\rho_{w}$) and between-study correlation ($\rho_{b}$), setting each to be either zero or strong (0.9). We perform simulations under a scenario in which the within-study treatment effects are the same for every study, and for a scenario in which within-study treatment effect variances vary across studies. We achieve the latter by keeping the between-participant outcome variances the same, and varying the sample sizes within studies. The full set of scenarios considered is provided in Supplementary Table B1.

*Outcome data generation* We start by simulating true treatment effects for two continuous outcomes using a bivariate normal distribution:

$\left(\begin{matrix} \theta_{1s} \\ \theta_{2s} \end{matrix}\right)\sim MVN\left(\left(\begin{matrix} \theta_{1} \\ \theta_{2} \end{matrix}\right),\Omega\right)$ with $\Omega=\left[\begin{matrix} \tau_{11}^{2} & \rho_{b}\tau_{11}\tau_{22} \\ \rho_{b}\tau_{11}\tau_{22} & \tau_{22}^{2} \end{matrix}\right]$.

with parameter values as specified above. Within each study, we simulate individual outcome data for each participant *i*, centering the control group participants on the observed control group mean across acute stroke trials for outcome 1 (the means and standard deviations for outcome 2 are arbitrary). The treatment group means are obtained by applying the simulated treatment effect for the study:

$\left(\begin{matrix} y_{1csi} \\ y_{2csi} \end{matrix}\right)\sim MVN\left(\left(\begin{matrix} \mu_{1c} \\ \mu_{2c} \end{matrix}\right),\mathbf{\Sigma}\right)$, $\left(\begin{matrix} y_{1tsi} \\ y_{2tsi} \end{matrix}\right)\sim MVN\left(\left(\begin{matrix} \mu_{1c}+\theta_{1s} \\ \mu_{2c}+\theta_{2s} \end{matrix}\right),\mathbf{\Sigma}\right)$ with $\mathbf{\Sigma}=\left[\begin{matrix} \sigma_{1}^{2} & \rho_{w}\sigma_{1}\sigma_{2} \\ \rho_{w}\sigma_{1}\sigma_{2} & \sigma_{2}^{2} \end{matrix}\right]$.

We then dichotomize each $y_{2tsi}$ and $y_{2csi}$ using cut-points $c_{t}$ and $c_{c}$ which are chosen so that $\Pr(y_{2tsi}\geq c_{t})=p_{t}$ and $\Pr(y_{2csi}\geq c_{c})=p_{c}$ with $p_{t}$ and $p_{c}$ the observed overall probabilities of death in the acute stroke data. These cutpoints define true odds ratios that are the same in every study, reflecting the lack of heterogeneity observed in the acute stroke data. The numbers of observations lying beyond the cut point are given by $r_{ts}$ and $r_{cs}$ in the two arms.

*Effect size calculation* We calculate the effect sizes and the associated variances for study *s* by

$$\begin{matrix} MD_{s}\text{ }=\text{ }\bar{y}_{1ts}-\bar{y}_{1cs},\text{ }\mathrm{var}(MD_{s})=\left(\frac{1}{N_{t}}+\frac{1}{N_{c}}\right)\sigma_{1}^{2} \\ \ln OR_{s}\text{ }=\text{ }\ln\left(\frac{r_{ts}/(N_{t}-r_{ts})}{r_{cs}/(N_{c}-r_{cs})}\right),\text{ }\mathrm{var}(\ln OR_{s})\text{ }=\text{ }\frac{1}{r_{ts}}+\frac{1}{N_{t}-r_{ts}}+\frac{1}{r_{cs}}+\frac{1}{N_{c}-r_{cs}} \end{matrix}$$

for mean difference and log odds ratio, respectively.

*Fitted models* We fit four models to the simulated data.

MU [Univariate meta-analyses]: Standard univariate meta-analyses are performed for the two outcomes separately.

MM(0) [Multivariate meta-analysis assuming zero within-study correlation): Within-study correlation, $\rho_{w}$, is assumed to be zero.

MM($\rho_{e}$) [Multivariate meta-analysis with a common within-study covariance for treatment effects for every study]: We calculate the within-study covariance between effect sizes as

$\mathrm{cov}(\text{MD},\log OR)=\rho_{e}\sqrt{\mathrm{var}(MD)\mathrm{var}(\log OR)}$.

MM($\rho_{e}$) [Multivariate meta-analysis with within-study covariances estimated from between-outcome correlations based on our formulae]: We calculate within-study covariance as

$$\mathrm{cov}(\text{MD},\log OR)=\rho_{o}\sigma_{1}\frac{1}{\sqrt{n_{c}}}\sqrt{\frac{1}{r_{c}}+\frac{1}{n_{c}-r_{c}}}+\rho_{o}\sigma_{1}\frac{1}{\sqrt{n_{t}}}\sqrt{\frac{1}{r_{t}}+\frac{1}{n_{t}-r_{t}}}$$

as in formula (1.3) of Table 1.

For the latter two methods, both correlation between effect sizes $\rho_{e}$ and correlation between outcomes $\rho_{o}$ are obtained from a pre-simulation run, and are kept fixed in the estimation. Correlation between outcome$\rho_{o}$ is invariant to sample size (number of participants in a study), while $\rho_{e}$ can vary with sample size (see Supplementary Table B1).

*Estimation process* We report the average bias for the estimated treatment effects $\hat{\theta}_{1}=MD$ and $\hat{\theta}_{2}=\ln OR$ and the elements of the variance-covariance matrix for these $\hat{\tau}_{11}^{2},\text{ }\hat{\tau}_{22}^{2}$ and $\hat{\tau}_{12}$ over 1000 Monte Carlo replications. We calculate the 95% confidence interval (CI) of $\hat{\theta}_{i}$ for the two outcomes using using $\hat{\theta}_{j}\pm t_{0.025,(n-1)}\sqrt{\mathrm{var}(\hat{\theta}_{j})};j=1,2$ where $t_{0.025,(n-1)}$ denote the 0.025 percentile of a *t*-distribution with $n-1$ degrees of freedom, with $n$ the total number of studies. This t-distribution is commonly used in the meta-analysis literature (Riley *et al.* 2007a, 2007b; Jackson *et al.* 2011; Ma & Mazumda 2011), although it is only an approximation (Riley *et al.* 2007b). We also calculated the proportion of the 95% CIs that cover the true $\theta_{j}$ *j=1,2*. We estimated the mean squared error $\text{MSE(}\hat{\theta}_{j}\text{)}$ across the 1000 simulations as $\sum_{k=1}^{1000} \left(\hat{\theta}_{jk}-\theta_{j}\right)^{2}/1000$. We carried out all data simulations and analyses using the software R (R Foundation for Statistical Computing, Vienna, Austria).

*Results* Summary statistics from the simulations are given in Supplementary Table B2 –B4. Our first observation is that treatment effects are well estimated by all methods, and there is little difference between univariate and multivariate approaches. We note that multivariate meta-analysis is most likely to offer advantages over a univariate approach when there are non-ignorable missing data (Kirkham *et al*. 2012). Our simulation study used complete case data and did not address this issue.

Comparing results across Tables B2-B4 shows the impact of the extent of heterogeneity for outcome two, with $\tau_{2}^{2}=0, 0.1^{2}, 0.2^{2}$, respectively. Table B2 shows that when $\tau_{2}^{2}=0$, the multivariate approach reduces bias in estimating $\tau_{1}$ but increase bias for $\tau_{2}$. It is therefore not clear whether a multivariate approach is better than a univariate approach when there is heterogeneity in one outcome but not in the other. However, when $\tau_{2}$ slightly departs from zero, improvement in estimates for between-study variance is evident in scenario 13-16,18,21-22 in Table B3. If we increase the $\tau_{2}$ to 0.2^2^ , there are consistent improvements in parameters estimates for between-study variance for both outcomes through all scenarios in Table B4. This suggests that a multivariate approach is likely to outperform a univariate approach when there is heterogeneity for both outcomes, but not necessarily otherwise.

We turn now to the impact of the magnitude of the correlation. Strong within-study correlation is assumed in scenarios 13-20 and 25-32. In these situations, both UM and MM(0) are misspecified models with respect to the within-study correlation. MM($\rho_{e}$) assumes a common correlation between treatment effects and MM($\rho_{o}$) assumes a common correlation between outcomes, using our formulae to approximate the within-study covariance. We expect that UM and MM(0) will be worse than the latter two with misspecification of the within-study correlation; while MM($\rho_{o}$) should be similar to MM($\rho_{e}$). We observe that bias for $\tau_{11}^{2}$ in UM and bias for $\tau_{22}^{2}$ in MM(0) are inflated in scenarios 13-20 and 25-32. In Table B3, the bias for between-study correlation $\rho_{b}$ in MM(0) is sometimes inflated, particularly in scenarios 13-14, and 17-18 where the between-study correlation is zero; the biases are 0.78, 0.92, 0.85 and 0.66, respectively. These biases appear to be serious, given the parameter space [-1, 1] for correlation coefficients. Similarly findings are given in scenarios 25-26, and 29-30. These indicate that when correlation is strong (weak) for within- (between-) study, then assuming zero within-study correlation will introduce bias into estimates for between-study correlation. This is one other situation in which correct specification or approximation of within-study correlation is important.

Estimation from MM($\rho_{o}$) is generally not worse than MM($\rho_{e}$). The particular situation in which we expect MM($\rho_{o}$) to perform better is when sample sizes vary across studies (the even-numbered scenarios), since the covariances between treatment effects then vary across studies even if the covariances between outcomes remain the same. We observe such a pattern for situations in which between-study correlation is low, but not when between-study correlation is high (which might be explained in part by our relatively small underlying heterogeneity variance for the second outcome, which does not allow the high between-study correlation to manifest itself).

Finally, Tables B3 and B4 show that, if there are large number of studies (n=50), the bias in the between-study variance estimate is reduced by using a multivariate approach, and the bias in the between-study correlation estimate is minimized in our proposed approach. However, when there are a few studies (n=10), no clear improvement are observed in the multivariate approach over the univariate approach, unless the within- (between-) study correlations are high (low).

**References for supplementary material B**

Jackson D, White IR, Thompson SG. Extending DerSimonian and Laird's methodology to perform multivariate random effects meta-analyses. *Stat.Med.* 2010; **29**(12):1282-1297.

Riley RD, Abrams KR, Lambert PC, Sutton AJ, Thompson JR. An evaluation of bivariate random-effects meta-analysis for the joint synthesis of two correlated outcomes. *Stat.Med.* 2007; **26**(1):78-97.

Riley RD, Abrams KR, Sutton AJ, Lambert PC, Thompson JR. Bivariate random-effects meta-analysis and the estimation of between-study correlation. *BMC.Med.Res.Methodol.* 2007; **7**:3.

Ma Y, Mazumdar M. Multivariate meta-analysis: a robust approach based on the theory of U-statistic. *Stat.Med.* 2011; **30**(24):2911-2929.

Kirkham JJ, Riley RD, Williamson PR. A multivariate meta-analysis approach for reducing the impact of outcome reporting bias in systematic reviews. *Stat.Med.* 2012; **31**(20):2179-2195.

**Supplementary Table B1** Correlation between effect size and outcomes based on data simulated from bivariate normal distribution with within- and between study correlation $\rho_{w}$ and $\rho_{b}$. Scenario numbers appear in square brackets.

| Pre-specified $\rho_{w}$ | Pre-specified $\rho_{b}$ | $\rho_{o}$ based on 1000 Monte-Carlo simulation | $\rho_{e}$ based on variable sample size | | | | | |
| --- | --- | --- | --- | --- | --- | --- | --- | --- |
|  |  |  | $n_{s}=50\text{ }$ | | | $n_{s}=10\text{ }$ | | |
|  |  |  | $n_{t}(n_{c})=200$ |  | U (20,200) | $n_{t}(n_{c})=20$ |  | U (20,200) |
| 0.9 | 0 | 0.667 | 0.30 [1] |  | 0.37 [2] | 0.45 [5] |  | 0.27 [6] |
| 0.9 | 0.9 | 0.668 | 0.52 [3] |  | 0.55 [4] | 0.50 [7] |  | 0.59 [8] |
| 0 | 0 | 0.002 | 0.05 [9] |  | - | -0.03 [11] |  | - |
| 0 | 0.9 | 0.016 | 0.26 [10] |  | - | 0.032 [12] |  | - |
| $\rho_{b}$ = between-study correlations; $\rho_{w}$= within-study correlations; $\rho_{o}$= Estimated within-study correlations between outcomes; $\rho_{e}$= Estimated within-study correlations between treatment effects, given sample sizes; $n_{s}$=Number of studies; $n_{t}(n_{c})$= Number of participants randomized in treatment (control) groups. | | | | | | | | |

| **Supplementary Table B2.** Simulation results over 1000 Monte Carlo replications. | | | | | | | | | |
| --- | --- | --- | --- | --- | --- | --- | --- | --- | --- |
| Methods | Bias-M $\hat{\beta}_{1}$ | Bias-M $\hat{\beta}_{2}$ | MSE $\hat{\beta}_{1}$ | MSE $\hat{\beta}_{2}$ | t coverage $\hat{\beta}_{1}$ | t coverage $\hat{\beta}_{2}$ | Bias-M $\hat{\tau}_{1}$ | Bias-M $\hat{\tau}_{2}$ | Bias-M $\hat{\rho}_{12}$ |
| Scenario 1: $n_{s}=50,\text{ }n_{t}=n_{c}=200,\text{ }\rho_{w}=0.9,\text{ }\rho_{b}=0.$ | | | | | | | | | |
| UM | 0.009 | -0.009 | 0.450 | 0.001 | 0.949 | 0.943 | -0.016 | 0.020 | NA |
| MM(0) | 0.009 | -0.009 | 0.450 | 0.001 | 0.949 | 0.947 | -0.014 | 0.059 | 0.887 |
| MM($\rho_{e}$) | 0.009 | -0.009 | 0.450 | 0.001 | 0.949 | 0.944 | -0.014 | 0.043 | 0.634 |
| MM($\rho_{o}$) | 0.008 | -0.009 | 0.450 | 0.001 | 0.949 | 0.947 | 0.003 | 0.035 | 0.005 |
| Scenario 2: $n_{s}=50,\text{ }n_{t}=n_{c}\sim U(20,\text{ }200),\text{ }\rho_{w}=0.9,\text{ }\rho_{b}=0.$ | | | | | | | | | |
| UM | -0.009 | -0.013 | 0.504 | 0.002 | 0.944 | 0.947 | -0.046 | 0.025 | NA |
| MM(0) | -0.009 | -0.012 | 0.509 | 0.002 | 0.947 | 0.955 | -0.012 | 0.097 | 0.953 |
| MM($\rho_{e}$) | -0.010 | -0.013 | 0.500 | 0.002 | 0.940 | 0.948 | -0.053 | 0.057 | 0.631 |
| MM($\rho_{o}$) | -0.010 | -0.013 | 0.498 | 0.002 | 0.945 | 0.949 | -0.012 | 0.046 | 0.101 |
| Scenario 3: $n_{s}=50,\text{ }n_{t}=n_{c}=200,\text{ }\rho_{w}=0.9,\text{ }\rho_{b}=0.9.$ | | | | | | | | | |
| UM | 0.009 | -0.009 | 0.450 | 0.001 | 0.949 | 0.944 | -0.016 | 0.020 | NA |
| MM(0) | 0.009 | -0.009 | 0.450 | 0.001 | 0.949 | 0.949 | -0.013 | 0.068 | 0.041 |
| MM($\rho_{e}$) | 0.009 | -0.009 | 0.450 | 0.001 | 0.949 | 0.948 | -0.014 | 0.048 | -0.143 |
| MM($\rho_{o}$) | 0.009 | -0.009 | 0.450 | 0.001 | 0.949 | 0.947 | 0.002 | 0.035 | -0.595 |
| Scenario 4: $n_{s}=50,\text{ }n_{t}=n_{c}\sim U(20,\text{ }200),\text{ }\rho_{w}=0.9,\text{ }\rho_{b}=0.9.$ | | | | | | | | | |
| UM | -0.009 | -0.013 | 0.504 | 0.002 | 0.944 | 0.946 | -0.046 | 0.026 | NA |
| MM(0) | -0.009 | -0.012 | 0.509 | 0.002 | 0.948 | 0.959 | -0.006 | 0.107 | 0.075 |
| MM($\rho_{e}$) | -0.009 | -0.012 | 0.500 | 0.002 | 0.940 | 0.954 | -0.051 | 0.061 | -0.190 |
| MM($\rho_{o}$) | -0.009 | -0.012 | 0.499 | 0.002 | 0.944 | 0.951 | -0.014 | 0.047 | -0.587 |
| Scenario 5: $n_{s}=10,\text{ }n_{t}=n_{c}=20,\text{ }\rho_{w}=0.9,\text{ }\rho_{b}=0.$ | | | | | | | | | |
| UM | -0.147 | -0.025 | 5.717 | 0.050 | 0.967 | 0.989 | -0.734 | 0.089 | NA |
| MM(0) | -0.147 | -0.025 | 5.717 | 0.052 | 0.979 | 0.992 | 0.219 | 0.333 | 0.848 |
| MM($\rho_{e}$) | -0.154 | -0.026 | 5.609 | 0.051 | 0.970 | 0.990 | -0.408 | 0.223 | 0.516 |
| MM($\rho_{o}$) | -0.157 | -0.026 | 5.581 | 0.050 | 0.969 | 0.990 | -0.100 | 0.218 | 0.193 |
| Scenario 6: $n_{s}=10,\text{ }n_{t}=n_{c}\sim U(20,\text{ }200),\text{ }\rho_{w}=0.9,\text{ }\rho_{b}=0.$ | | | | | | | | | |
| UM | -0.049 | -0.015 | 2.720 | 0.012 | 0.953 | 0.976 | -0.367 | 0.053 | NA |
| MM(0) | -0.048 | -0.015 | 2.757 | 0.012 | 0.956 | 0.983 | -0.252 | 0.134 | 0.685 |
| MM($\rho_{e}$) | -0.048 | -0.015 | 2.698 | 0.012 | 0.956 | 0.983 | -0.306 | 0.113 | 0.451 |
| MM($\rho_{o}$) | -0.046 | -0.015 | 2.632 | 0.012 | 0.961 | 0.983 | -0.170 | 0.106 | 0.065 |
| Scenario 7: $n_{s}=10,\text{ }n_{t}=n_{c}=20,\text{ }\rho_{w}=0.9,\text{ }\rho_{b}=0.9.$ | | | | | | | | | |
| UM | -0.147 | -0.026 | 5.717 | 0.050 | 0.967 | 0.988 | -0.734 | 0.090 | NA |
| MM(0) | -0.147 | -0.025 | 5.717 | 0.052 | 0.978 | 0.991 | 0.242 | 0.337 | -0.046 |
| MM($\rho_{e}$) | -0.151 | -0.026 | 5.602 | 0.051 | 0.969 | 0.990 | -0.420 | 0.221 | -0.380 |
| MM($\rho_{o}$) | -0.153 | -0.026 | 5.577 | 0.050 | 0.968 | 0.989 | -0.128 | 0.216 | -0.685 |
| Scenario 8: $n_{s}=10,\text{ }n_{t}=n_{c}\sim U(20,\text{ }200),\text{ }\rho_{w}=0.9,\text{ }\rho_{b}=0.9.$ | | | | | | | | | |
| UM | -0.049 | -0.016 | 2.720 | 0.012 | 0.953 | 0.981 | -0.367 | 0.053 | NA |
| MM(0) | -0.048 | -0.015 | 2.758 | 0.012 | 0.957 | 0.985 | -0.245 | 0.139 | -0.147 |
| MM($\rho_{e}$) | -0.047 | -0.015 | 2.699 | 0.012 | 0.956 | 0.983 | -0.308 | 0.120 | -0.384 |
| MM($\rho_{o}$) | -0.045 | -0.016 | 2.636 | 0.012 | 0.959 | 0.983 | -0.176 | 0.106 | -0.724 |
| Scenario 9: $n_{s}=50,\text{ }n_{t}=n_{c}=200,\text{ }\rho_{w}=\rho_{b}=0.$ | | | | | | | | | |
| UM | 0.009 | -0.010 | 0.450 | 0.001 | 0.949 | 0.952 | -0.016 | 0.021 | NA |
| MM(0) | 0.009 | -0.010 | 0.450 | 0.001 | 0.949 | 0.952 | -0.016 | 0.0348 | 0.019 |
| MM($\rho_{e}$) | 0.009 | -0.010 | 0.450 | 0.001 | 0.949 | 0.952 | -0.016 | 0.0349 | -0.024 |
| MM($\rho_{o}$) | 0.009 | -0.010 | 0.450 | 0.001 | 0.949 | 0.952 | -0.016 | 0.0349 | 0.015 |
| Scenario 10: $n_{s}=50,\text{ }n_{t}=n_{c}=200,\text{ }\rho_{w}=0,\text{ }\rho_{b}=0.9.$ | | | | | | | | | |
| UM | 0.009 | -0.011 | 0.450 | 0.001 | 0.949 | 0.948 | -0.016 | 0.021 | NA |
| MM(0) | 0.009 | -0.011 | 0.450 | 0.001 | 0.949 | 0.951 | -0.016 | 0.036 | -0.639 |
| MM($\rho_{e}$) | 0.009 | -0.011 | 0.450 | 0.001 | 0.949 | 0.951 | -0.016 | 0.035 | -0.751 |
| MM($\rho_{o}$) | 0.009 | -0.011 | 0.445 | 0.001 | 0.949 | 0.951 | -0.016 | 0.036 | -0.660 |
| Scenario 11: $n_{s}=10,\text{ }n_{t}=n_{c}=20,\text{ }\rho_{w}=\rho_{b}=0.$ | | | | | | | | | |
| UM | -0.147 | -0.022 | 5.717 | 0.051 | 0.967 | 0.99 | -0.734 | 0.094 | NA |
| MM(0) | -0.147 | -0.022 | 5.717 | 0.051 | 0.971 | 0.991 | -0.318 | 0.216 | -0.039 |
| MM($\rho_{e}$) | -0.147 | -0.022 | 5.717 | 0.051 | 0.970 | 0.991 | -0.316 | 0.216 | -0.056 |
| MM($\rho_{o}$) | -0.147 | -0.022 | 5.717 | 0.051 | 0.971 | 0.991 | -0.318 | 0.216 | -0.040 |
| Scenario 12: $n_{s}=10,\text{ }n_{t}=n_{c}=20,\text{ }\rho_{w}=0,\text{ }\rho_{b}=0.9.$ | | | | | | | | | |
| UM | -0.147 | -0.023 | 5.717 | 0.051 | 0.967 | 0.989 | -0.734 | 0.095 | NA |
| MM(0) | -0.147 | -0.022 | 5.717 | 0.052 | 0.972 | 0.991 | -0.319 | 0.216 | -0.917 |
| MM($\rho_{e}$) | -0.147 | -0.022 | 5.717 | 0.052 | 0.972 | 0.991 | -0.318 | 0.216 | -0.950 |
| MM($\rho_{o}$) | -0.147 | -0.022 | 5.717 | 0.0519 | 0.972 | 0.991 | -0.320 | 0.216 | -0.938 |
| UM: multiple univariate meta-analyses; MM(0): multivariate meta-analyses; MM0-assuming zero as within-study correlations; MM($\rho_{e}$)-assuming common non-zero within-study correlations between treatment effects; MM($\rho_{o}$)-assuming common non-zero within-study correlations between outcomes. $n_{t}$ and $n_{c}$ number of participants in treatment group and control group, respectively; $n_{s}$ number of studies; $\tau_{1}$ and $\tau_{2}$ between-study standard deviation for treatment group and control group, respectively; $\rho_{\text{b}}$ between-study correlation coefficients for overall effects; $\rho_{\text{w}}$ within-study correlation coefficients for outcomes (before dichotomized); $p_{t}$ and $p_{c}$ event rates in treatment and control group, respectively. | | | | | | | | | |

| **Supplementary Table B3.** Simulation results over 1000 Monte Carlo replications. | | | | | | | | | |
| --- | --- | --- | --- | --- | --- | --- | --- | --- | --- |
| Methods | Bias-M $\hat{\beta}_{1}$ | Bias-M $\hat{\beta}_{2}$ | MSE $\hat{\beta}_{1}$ | MSE $\hat{\beta}_{2}$ | t coverage $\hat{\beta}_{1}$ | t coverage $\hat{\beta}_{2}$ | Bias-M $\hat{\tau}_{1}$ | Bias-M $\hat{\tau}_{2}$ | Bias-M $\hat{\rho}_{12}$ |
| Scenario 13: $n_{s}=50,\text{ }n_{t}=n_{c}=200,\text{ }\rho_{w}=0.9,\text{ }\rho_{b}=0.$ | | | | | | | | | |
| UM | 0.009 | -0.012 | 0.450 | 0.001 | 0.949 | 0.933 | -0.016 | -0.030 | NA |
| MM(0) | 0.009 | -0.012 | 0.450 | 0.001 | 0.949 | 0.940 | -0.015 | -0.007 | 0.783 |
| MM($\rho_{e}$) | 0.007 | -0.012 | 0.450 | 0.001 | 0.949 | 0.935 | -0.015 | -0.017 | 0.482 |
| MM($\rho_{o}$) | 0.004 | -0.012 | 0.450 | 0.001 | 0.949 | 0.933 | -0.007 | -0.020 | -0.068 |
| Scenario 14: $n_{s}=50,\text{ }n_{t}=n_{c}\sim U(20,\text{ }200),\text{ }\rho_{w}=0.9,\text{ }\rho_{b}=0.$ | | | | | | | | | |
| UM | -0.009 | -0.015 | 0.504 | 0.002 | 0.944 | 0.944 | -0.046 | -0.033 | NA |
| MM(0) | -0.009 | -0.015 | 0.509 | 0.002 | 0.946 | 0.952 | -0.014 | 0.025 | 0.915 |
| MM($\rho_{e}$) | -0.012 | -0.015 | 0.499 | 0.002 | 0.943 | 0.944 | -0.056 | -0.011 | 0.489 |
| MM($\rho_{o}$) | -0.014 | -0.015 | 0.498 | 0.002 | 0.943 | 0.946 | -0.022 | -0.016 | 0.017 |
| Scenario 15: $n_{s}=50,\text{ }n_{t}=n_{c}=200,\text{ }\rho_{w}=0.9,\text{ }\rho_{b}=0.9.$ | | | | | | | | | |
| UM | 0.009 | -0.012 | 0.450 | 0.001 | 0.949 | 0.934 | -0.016 | -0.030 | NA |
| MM(0) | 0.009 | -0.010 | 0.450 | 0.001 | 0.949 | 0.955 | -0.005 | 0.038 | 0.094 |
| MM($\rho_{e}$) | 0.010 | -0.010 | 0.450 | 0.001 | 0.948 | 0.950 | -0.012 | 0.003 | 0.009 |
| MM($\rho_{o}$) | 0.010 | -0.010 | 0.450 | 0.001 | 0.948 | 0.946 | -0.006 | -0.006 | -0.067 |
| Scenario 16: $n_{s}=50,\text{ }n_{t}=n_{c}\sim U(20,\text{ }200),\text{ }\rho_{w}=0.9,\text{ }\rho_{b}=0.9.$ | | | | | | | | | |
| UM | -0.009 | -0.015 | 0.504 | 0.002 | 0.944 | 0.946 | -0.046 | -0.031 | NA |
| MM(0) | -0.009 | -0.013 | 0.510 | 0.002 | 0.948 | 0.968 | 0.022 | 0.074 | 0.098 |
| MM($\rho_{e}$) | -0.007 | -0.013 | 0.499 | 0.002 | 0.940 | 0.952 | -0.041 | 0.008 | -0.051 |
| MM($\rho_{o}$) | -0.007 | -0.013 | 0.499 | 0.002 | 0.943 | 0.949 | -0.028 | 0.001 | -0.111 |
| Scenario 17: $n_{s}=10,\text{ }n_{t}=n_{c}=20,\text{ }\rho_{w}=0.9,\text{ }\rho_{b}=0.$ | | | | | | | | | |
| UM | -0.147 | -0.025 | 5.717 | 0.051 | 0.967 | 0.988 | -0.734 | 0.012 | NA |
| MM(0) | -0.147 | -0.025 | 5.717 | 0.053 | 0.979 | 0.991 | 0.212 | 0.255 | 0.847 |
| MM($\rho_{e}$) | -0.157 | -0.026 | 5.610 | 0.052 | 0.970 | 0.989 | -0.412 | 0.145 | 0.514 |
| MM($\rho_{o}$) | -0.163 | -0.027 | 5.578 | 0.051 | 0.969 | 0.987 | -0.106 | 0.139 | 0.185 |
| Scenario 18: $n_{s}=10,\text{ }n_{t}=n_{c}\sim U(20,\text{ }200),\text{ }\rho_{w}=0.9,\text{ }\rho_{b}=0.$ | | | | | | | | | |
| UM | -0.049 | -0.018 | 2.720 | 0.013 | 0.953 | 0.973 | -0.367 | -0.015 | NA |
| MM(0) | -0.048 | -0.017 | 2.753 | 0.013 | 0.955 | 0.980 | -0.252 | 0.061 | 0.657 |
| MM($\rho_{e}$) | -0.049 | -0.018 | 2.692 | 0.013 | 0.956 | 0.979 | -0.301 | 0.041 | 0.375 |
| MM($\rho_{o}$) | -0.049 | -0.018 | 2.639 | 0.013 | 0.959 | 0.978 | -0.173 | 0.037 | 0.009 |
| Scenario 19: $n_{s}=10,\text{ }n_{t}=n_{c}=20,\text{ }\rho_{w}=0.9,\text{ }\rho_{b}=0.9.$ | | | | | | | | | |
| UM | -0.147 | -0.027 | 5.717 | 0.051 | 0.967 | 0.984 | -0.734 | 0.015 | NA |
| MM(0) | -0.147 | -0.025 | 5.717 | 0.052 | 0.979 | 0.990 | 0.318 | 0.277 | -0.034 |
| MM($\rho_{e}$) | -0.147 | -0.025 | 5.583 | 0.051 | 0.969 | 0.986 | -0.443 | 0.152 | -0.321 |
| MM($\rho_{o}$) | -0.144 | -0.025 | 5.559 | 0.051 | 0.969 | 0.988 | -0.218 | 0.142 | -0.570 |
| Scenario 20: $n_{s}=10,\text{ }n_{t}=n_{c}\sim U(20,\text{ }200),\text{ }\rho_{w}=0.9,\text{ }\rho_{b}=0.9.$ | | | | | | | | | |
| UM | -0.049 | -0.017 | 2.720 | 0.012 | 0.953 | 0.981 | -0.367 | -0.015 | NA |
| MM(0) | -0.048 | -0.016 | 2.762 | 0.013 | 0.957 | 0.988 | -0.210 | 0.089 | -0.026 |
| MM($\rho_{e}$) | -0.047 | -0.016 | 2.691 | 0.012 | 0.956 | 0.985 | -0.305 | 0.054 | -0.231 |
| MM($\rho_{o}$) | -0.044 | -0.016 | 2.655 | 0.012 | 0.959 | 0.983 | -0.211 | 0.042 | -0.448 |
| Scenario 21: $n_{s}=50,\text{ }n_{t}=n_{c}=200,\text{ }\rho_{w}=\rho_{b}=0.$ | | | | | | | | | |
| UM | 0.009 | -0.013 | 0.450 | 0.001 | 0.949 | 0.945 | -0.016 | -0.029 | NA |
| MM(0) | 0.009 | -0.013 | 0.450 | 0.001 | 0.949 | 0.946 | -0.016 | -0.021 | 0.040 |
| MM($\rho_{e}$) | 0.009 | -0.013 | 0.450 | 0.001 | 0.949 | 0.945 | -0.016 | -0.021 | -0.050 |
| MM($\rho_{o}$) | 0.009 | -0.013 | 0.450 | 0.001 | 0.949 | 0.946 | -0.016 | -0.021 | 0.037 |
| Scenario 22: $n_{s}=50,\text{ }n_{t}=n_{c}=200,\text{ }\rho_{w}=0,\text{ }\rho_{b}=0.9.$ | | | | | | | | | |
| UM | 0.009 | -0.013 | 0.450 | 0.001 | 0.949 | 0.935 | -0.016 | -0.028 | NA |
| MM(0) | 0.009 | -0.012 | 0.450 | 0.001 | 0.949 | 0.944 | -0.015 | -0.004 | -0.107 |
| MM($\rho_{e}$) | 0.008 | -0.012 | 0.450 | 0.001 | 0.949 | 0.940 | -0.016 | -0.014 | -0.324 |
| MM($\rho_{o}$) | 0.009 | -0.012 | 0.450 | 0.001 | 0.949 | 0.944 | -0.015 | -0.005 | -0.118 |
| Scenario 23: $n_{s}=10,\text{ }n_{t}=n_{c}=20,\text{ }\rho_{w}=\rho_{b}=0.$ | | | | | | | | | |
| UM | -0.147 | -0.022 | 5.717 | 0.051 | 0.967 | 0.990 | -0.734 | 0.021 | NA |
| MM(0) | -0.147 | -0.021 | 5.717 | 0.052 | 0.970 | 0.992 | -0.319 | 0.141 | -0.052 |
| MM($\rho_{e}$) | -0.146 | -0.021 | 5.715 | 0.052 | 0.969 | 0.992 | -0.310 | 0.140 | -0.007 |
| MM($\rho_{o}$) | -0.147 | -0.021 | 5.717 | 0.052 | 0.970 | 0.992 | -0.317 | 0.141 | -0.055 |
| Scenario 24: $n_{s}=10,\text{ }n_{t}=n_{c}=20,\text{ }\rho_{w}=0,\text{ }\rho_{b}=0.9.$ | | | | | | | | | |
| UM | -0.147 | -0.024 | 5.717 | 0.051 | 0.967 | 0.991 | -0.734 | 0.020 | NA |
| MM(0) | -0.147 | -0.023 | 5.717 | 0.052 | 0.972 | 0.992 | -0.304 | 0.142 | -0.826 |
| MM($\rho_{e}$) | -0.148 | -0.024 | 5.717 | 0.052 | 0.972 | 0.992 | -0.311 | 0.142 | -0.872 |
| MM($\rho_{o}$) | -0.147 | -0.024 | 5.717 | 0.052 | 0.972 | 0.992 | -0.309 | 0.143 | -0.851 |
| UM: multiple univariate meta-analyses; MM(0): multivariate meta-analyses; MM0-assuming zero as within-study correlations; MM($\rho_{e}$)-assuming common non-zero within-study correlations between treatment effects; MM($\rho_{o}$)-assuming common non-zero within-study correlations between outcomes. $n_{t}$ and $n_{c}$ number of participants in treatment group and control group, respectively; $n_{s}$ number of studies; $\tau_{1}$ and $\tau_{2}$ between-study standard deviation for treatment group and control group, respectively; $\rho_{\text{b}}$ between-study correlation coefficients for overall effects; $\rho_{\text{w}}$ within-study correlation coefficients for outcomes (before dichotomized); $p_{t}$ and $p_{c}$ event rates in treatment and control group, respectively. | | | | | | | | | |

| **Supplementary Table B4.** Simulation results over 1000 Monte Carlo replications. | | | | | | | | | |
| --- | --- | --- | --- | --- | --- | --- | --- | --- | --- |
| Methods | Bias-M $\hat{\beta}_{1}$ | Bias-M $\hat{\beta}_{2}$ | MSE $\hat{\beta}_{1}$ | MSE $\hat{\beta}_{2}$ | t coverage $\hat{\beta}_{1}$ | t coverage $\hat{\beta}_{2}$ | Bias-M $\hat{\tau}_{1}$ | Bias-M $\hat{\tau}_{2}$ | Bias-M $\hat{\rho}_{12}$ |
| Scenario 25: $n_{s}=50,\text{ }n_{t}=n_{c}=200,\text{ }\rho_{w}=0.9,\text{ }\rho_{b}=0.$ | | | | | | | | | |
| UM | 0.009 | -0.018 | 0.450 | 0.002 | 0.949 | 0.925 | -0.016 | -0.044 | NA |
| MM(0) | 0.009 | -0.019 | 0.450 | 0.002 | 0.949 | 0.926 | -0.016 | -0.042 | 0.461 |
| MM($\rho_{e}$) | 0.003 | -0.019 | 0.450 | 0.002 | 0.949 | 0.926 | -0.017 | -0.043 | 0.240 |
| MM($\rho_{o}$) | -0.005 | -0.018 | 0.450 | 0.002 | 0.948 | 0.926 | -0.016 | -0.043 | -0.062 |
| Scenario 26: $n_{s}=50,\text{ }n_{t}=n_{c}\sim U(20,\text{ }200),\text{ }\rho_{w}=0.9,\text{ }\rho_{b}=0.$ | | | | | | | | | |
| UM | -0.009 | -0.022 | 0.504 | 0.003 | 0.944 | 0.931 | -0.046 | -0.062 | NA |
| MM(0) | -0.010 | -0.022 | 0.509 | 0.003 | 0.945 | 0.939 | -0.019 | -0.036 | 0.745 |
| MM($\rho_{e}$) | -0.018 | -0.022 | 0.500 | 0.003 | 0.941 | 0.929 | -0.061 | -0.054 | 0.322 |
| MM($\rho_{o}$) | -0.023 | -0.022 | 0.498 | 0.003 | 0.942 | 0.928 | -0.041 | -0.055 | -0.080 |
| Scenario 27: $n_{s}=50,\text{ }n_{t}=n_{c}=200,\text{ }\rho_{w}=0.9,\text{ }\rho_{b}=0.9.$ | | | | | | | | | |
| UM | 0.009 | -0.019 | 0.450 | 0.002 | 0.9490 | 0.930 | -0.016 | -0.043 | NA |
| MM(0) | 0.009 | -0.015 | 0.450 | 0.002 | 0.9490 | 0.956 | 0.012 | 0.006 | 0.099 |
| MM($\rho_{e}$) | 0.007 | -0.016 | 0.449 | 0.002 | 0.9480 | 0.939 | -0.018 | -0.035 | -0.004 |
| MM($\rho_{o}$) | 0.008 | -0.016 | 0.449 | 0.002 | 0.9480 | 0.939 | -0.017 | -0.034 | 0.002 |
| Scenario 28: $n_{s}=50,\text{ }n_{t}=n_{c}\sim U(20,\text{ }200),\text{ }\rho_{w}=0.9,\text{ }\rho_{b}=0.9.$ | | | | | | | | | |
| UM | -0.009 | -0.022 | 0.504 | 0.003 | 0.944 | 0.932 | -0.046 | -0.058 | NA |
| MM(0) | -0.010 | -0.017 | 0.510 | 0.003 | 0.949 | 0.958 | 0.063 | 0.036 | 0.100 |
| MM($\rho_{e}$) | -0.008 | -0.018 | 0.500 | 0.003 | 0.943 | 0.943 | -0.038 | -0.039 | -0.026 |
| MM($\rho_{o}$) | -0.008 | -0.018 | 0.501 | 0.003 | 0.941 | 0.943 | -0.045 | -0.033 | 0.011 |
| Scenario 29: $n_{s}=10,\text{ }n_{t}=n_{c}=20,\text{ }\rho_{w}=0.9,\text{ }\rho_{b}=0.$ | | | | | | | | | |
| UM | -0.149 | -0.032 | 5.717 | 0.053 | 0.967 | 0.985 | -0.73 | -0.065 | NA |
| MM(0) | -0.147 | -0.031 | 5.717 | 0.055 | 0.977 | 0.989 | 0.221 | 0.168 | 0.846 |
| MM($\rho_{e}$) | -0.168 | -0.033 | 5.612 | 0.053 | 0.97 | 0.985 | -0.414 | 0.062 | 0.511 |
| MM($\rho_{o}$) | -0.179 | -0.033 | 5.575 | 0.053 | 0.969 | 0.983 | -0.120 | 0.055 | 0.138 |
| Scenario 30: $n_{s}=10,\text{ }n_{t}=n_{c}\sim U(20,\text{ }200),\text{ }\rho_{w}=0.9,\text{ }\rho_{b}=0.$ | | | | | | | | | |
| UM | -0.049 | -0.025 | 2.720 | 0.016 | 0.953 | 0.956 | -0.367 | -0.076 | NA |
| MM(0) | -0.048 | -0.024 | 2.750 | 0.016 | 0.955 | 0.968 | -0.250 | -0.013 | 0.565 |
| MM($\rho_{e}$) | -0.053 | -0.025 | 2.690 | 0.016 | 0.956 | 0.965 | -0.296 | -0.029 | 0.287 |
| MM($\rho_{o}$) | -0.058 | -0.025 | 2.645 | 0.016 | 0.959 | 0.96 | -0.193 | -0.030 | -0.040 |
| Scenario 31: $n_{s}=10,\text{ }n_{t}=n_{c}=20,\text{ }\rho_{w}=0.9,\text{ }\rho_{b}=0.9.$ | | | | | | | | | |
| UM | -0.147 | -0.036 | 5.717 | 0.053 | 0.967 | 0.982 | -0.73 | -0.071 | NA |
| MM(0) | -0.147 | -0.031 | 5.717 | 0.055 | 0.979 | 0.990 | 0.438 | 0.209 | -0.006 |
| MM($\rho_{e}$) | -0.147 | -0.031 | 5.564 | 0.053 | 0.970 | 0.986 | -0.482 | 0.071 | -0.266 |
| MM($\rho_{o}$) | -0.141 | -0.030 | 5.538 | 0.053 | 0.970 | 0.986 | -0.327 | 0.054 | -0.470 |
| Scenario 32: $n_{s}=10,\text{ }n_{t}=n_{c}\sim U(20,\text{ }200),\text{ }\rho_{w}=0.9,\text{ }\rho_{b}=0.9.$ | | | | | | | | | |
| UM | -0.049 | -0.024 | 2.720 | 0.015 | 0.953 | 0.967 | -0.367 | -0.073 | NA |
| MM(0) | -0.050 | -0.020 | 2.758 | 0.015 | 0.959 | 0.983 | -0.144 | 0.040 | 0.037 |
| MM($\rho_{e}$) | -0.046 | -0.020 | 2.683 | 0.015 | 0.956 | 0.975 | -0.297 | -0.011 | -0.176 |
| MM($\rho_{o}$) | -0.044 | -0.020 | 2.672 | 0.015 | 0.956 | 0.974 | -0.259 | -0.016 | -0.233 |
| Scenario 33: $n_{s}=50,\text{ }n_{t}=n_{c}=200,\text{ }\rho_{w}=\rho_{b}=0.$ | | | | | | | | | |
| UM | 0.009 | -0.020 | 0.450 | 0.002 | 0.949 | 0.915 | -0.016 | -0.043 | NA |
| MM(0) | 0.009 | -0.020 | 0.450 | 0.002 | 0.949 | 0.916 | -0.016 | -0.042 | 0.010 |
| MM($\rho_{e}$) | 0.008 | -0.020 | 0.450 | 0.002 | 0.949 | 0.915 | -0.016 | -0.042 | -0.060 |
| MM($\rho_{o}$) | 0.009 | -0.020 | 0.450 | 0.002 | 0.949 | 0.916 | -0.016 | -0.042 | 0.009 |
| Scenario 34: $n_{s}=50,\text{ }n_{t}=n_{c}=200,\text{ }\rho_{w}=0,\text{ }\rho_{b}=0.9.$ | | | | | | | | | |
| UM | 0.009 | -0.019 | 0.450 | 0.002 | 0.949 | 0.917 | -0.016 | -0.041 | NA |
| MM(0) | 0.009 | -0.017 | 0.4450 | 0.002 | 0.949 | 0.934 | -0.013 | -0.031 | -0.016 |
| MM($\rho_{e}$) | 0.005 | -0.018 | 0.449 | 0.002 | 0.949 | 0.928 | -0.018 | -0.039 | -0.278 |
| MM($\rho_{o}$) | 0.009 | -0.017 | 0.450 | 0.002 | 0.949 | 0.934 | -0.014 | -0.031 | -0.022 |
| Scenario 35: $n_{s}=10,\text{ }n_{t}=n_{c}=20,\text{ }\rho_{w}=\rho_{b}=0.$ | | | | | | | | | |
| UM | -0.147 | -0.030 | 5.717 | 0.052 | 0.967 | 0.991 | -0.734 | -0.066 | NA |
| MM(0) | -0.147 | -0.029 | 5.717 | 0.053 | 0.970 | 0.992 | -0.297 | 0.054 | -0.046 |
| MM($\rho_{e}$) | -0.144 | -0.029 | 5.713 | 0.053 | 0.971 | 0.992 | -0.285 | 0.054 | 0.049 |
| MM($\rho_{o}$) | -0.147 | -0.029 | 5.717 | 0.053 | 0.970 | 0.992 | -0.298 | 0.054 | -0.046 |
| Scenario 36: $n_{s}=10,\text{ }n_{t}=n_{c}=20,\text{ }\rho_{w}=0,\text{ }\rho_{b}=0.9.$ | | | | | | | | | |
| UM | -0.147 | -0.035 | 5.717 | 0.053 | 0.967 | 0.988 | -0.734 | -0.067 | NA |
| MM(0) | -0.147 | -0.034 | 5.717 | 0.054 | 0.972 | 0.991 | -0.280 | 0.056 | -0.730 |
| MM($\rho_{e}$) | -0.149 | -0.034 | 5.718 | 0.054 | 0.972 | 0.991 | -0.292 | 0.056 | -0.810 |
| MM($\rho_{o}$) | -0.147 | -0.034 | 5.717 | 0.054 | 0.973 | 0.991 | -0.285 | 0.056 | -0.752 |
| UM: multiple univariate meta-analyses; MM(0): multivariate meta-analyses; MM0-assuming zero as within-study correlations; MM($\rho_{e}$)-assuming common non-zero within-study correlations between treatment effects; MM($\rho_{o}$)-assuming common non-zero within-study correlations between outcomes. $n_{t}$ and $n_{c}$ number of participants in treatment group and control group, respectively; $n_{s}$ number of studies; $\tau_{1}$ and $\tau_{2}$ between-study standard deviation for treatment group and control group, respectively; $\rho_{\text{b}}$ between-study correlation coefficients for overall effects; $\rho_{\text{w}}$ within-study correlation coefficients for outcomes (before dichotomized); $p_{t}$ and $p_{c}$ event rates in treatment and control group, respectively. | | | | | | | | | |

| **Supplementary** **Figure B1** Approximation errors for continuous outcomes (mean squared error): formulae 1.2 (left) and 1.6 (right). Each line represents a simulation scenario according to variable number of participants $n_{t}=n_{c}=20\text{ (purple), }50\text{ (green), 100 (blue), 200 (red), 1000 (black).}$ |
| --- |

| **Supplementary** **Figure B2** Approximation errors for continuous and dichotomous outcomes (mean squared error): formulae 1.3 (top left), 1.4 (top middle), 1.5 (top right); and formulae 1.7 (bottom left), 1.8 (bottom middle) and 1.9 (bottom right), with sample size $n_{t}=n_{c}=20\text{ (purple), }50\text{ (green), 100 (blue), 200 (red), 1000 (black).}$ |
| --- |

| **Supplementary** **Figure B3** Approximation errors for dichotomous outcomes (mean squared error): formulae 1.10 (top left), 1.11 (top middle), 1.12 (top right); and formula 1.13 (bottom left), 1.14 (bottom middle) and 1.15 (bottom right), with sample size $n_{t}=n_{c}=20\text{ (purple), }50\text{ (green), 100 (blue), 200 (red), 1000 (black).}$ |
| --- |

**Supplementary material C**

**Supplementary Table C1** Data for SBP and DBP in trials of vasoactive drugs for acute stroke [4].

|  |  |  | SBP | | | | | |  | DBP | | | | | | |
| --- | --- | --- | --- | --- | --- | --- | --- | --- | --- | --- | --- | --- | --- | --- | --- | --- |
| Study | N_t | N_c | Treatment | | | Control | | |  | Treatment | | | Control | | | |
|  |  |  | N | Mean | SD | N | Mean | SD |  | N | Mean | SD | | N | Mean | SD |
| Barer 1988 atenolol | 18 | 11 | 18 | 138.06 | 24.68 | 10 | 140.53 | 23.27 |  | 18 | 77.61 | 11.34 | | 10 | 81.05 | 14.39 |
| Barer 1988 propanolol | 16 | 11 | 14 | 142.14 | 30.93 | 9 | 140.53 | 23.27 |  | 14 | 80.71 | 16.51 | | 9 | 81.05 | 14.39 |
| Barer 1988/50 mg | 102 | 50 | 95 | 137.31 | 25.14 | 47 | 145.47 | 22.58 |  | 95 | 79.63 | 13.62 | | 47 | 86.07 | 15.23 |
| Barer 1988/80 mg | 101 | 50 | 97 | 142.3 | 23.85 | 48 | 145.47 | 22.58 |  | 97 | 82.66 | 13.93 | | 48 | 86.07 | 15.23 |
| ASCLEPIOS 1990 | 120 | 114 | 107 | 144.73 | 23.77 | 97 | 144.88 | 23.96 |  | 107 | 80.98 | 11.83 | | 97 | 83.37 | 13.5 |
| Limburg 1990 | 12 | 14 | 10 | 141 | 26.65 | 6 | 150.83 | 24.58 |  | 10 | 86.1 | 18.53 | | 6 | 84.17 | 13.57 |
| Norris 1994 | 96 | 93 | 83 | 133.62 | 16.3 | 75 | 149.87 | 25.1 |  | 83 | 71.41 | 10.33 | | 75 | 83.29 | 12.42 |
| Bogousslavsky 1990 | 24 | 28 | 24 | 134 | 24 | 28 | 141 | 16 |  | 24 | 81 | 11 | | 28 | 80 | 11 |
| Kaste 1994/120 mg | 176 | 174 | 160 | 146.6 | 25.81 | 163 | 148.5 | 25.81 |  | 160 | 84.3 | 14.84 | | 161 | 89.2 | 14.24 |
| Lowe 1993 | 56 | 56 | 54 | 145.19 | 24.65 | 54 | 141.02 | 22.39 |  | 54 | 85.11 | 13.29 | | 54 | 81.3 | 11.33 |
| Paci 1989/120 mg | 19 | 22 | 19 | 136.7 | 17.4 | 22 | 145.7 | 19.7 |  | 19 | 82.8 | 10 | | 22 | 85.2 | 9.8 |
| Squire 1996 | 75 | 72 | 68 | 145.72 | 22.65 | 55 | 147.85 | 21.82 |  | 68 | 81.35 | 12.66 | | 55 | 82.53 | 11.26 |
| VENUS 1995 | 225 | 229 | 215 | 152.69 | 25.31 | 213 | 152.16 | 26.5 |  | 215 | 84.16 | 12.81 | | 213 | 85.81 | 13.54 |
| Lees 1995 | 30 | 30 | 27 | 147.48 | 28.27 | 25 | 139.52 | 23.82 |  | 27 | 79.63 | 17.24 | | 25 | 76.92 | 15.79 |
| IMAGES Pilot | 26 | 25 | 26 | 147.35 | 17.54 | 25 | 155.94 | 27.31 |  | 26 | 72.94 | 10.12 | | 25 | 82.12 | 10.9 |
| Muir 1995 | 19 | 6 | 15 | 150.93 | 20.33 | 4 | 159.25 | 22.02 |  | 15 | 82.2 | 15.49 | | 4 | 89.75 | 15.11 |
| Strand 1984 | 13 | 13 | 13 | 168.46 | 35.73 | 12 | 150.42 | 18.4 |  | 13 | 84.62 | 15.87 | | 12 | 80.83 | 10.19 |
| PRISTINE | 313 | 307 | 310 | 155.7 | 24.45 | 307 | 154.33 | 24.95 |  | 310 | 87.8 | 12.55 | | 307 | 85.96 | 12.64 |
| Steiner 1986 | 55 | 45 | 44 | 139.89 | 20.01 | 37 | 147.3 | 26.21 |  | 44 | 82.73 | 10.37 | | 37 | 86.89 | 15.56 |
| Herrschaft 1988 | 24 | 20 | 23 | 176.6 | 19.6 | 17 | 174.2 | 20.2 |  | 23 | 94.5 | 12.4 | | 17 | 95.2 | 10.2 |
| Huczynski 1988 | 15 | 15 | 15 | 140.4 | 27.84 | 15 | 146.33 | 26.94 |  | 15 | 87 | 11.19 | | 15 | 86.47 | 17.31 |

**Supplementary Table C2** Data for outcomes of death and ‘death and disability’ in trials of vasoactive drugs for acute stroke [4].

|  |  |  | Death | | | |  | Death or disability | | | |
| --- | --- | --- | --- | --- | --- | --- | --- | --- | --- | --- | --- |
| Study | N_t | N_c | Treatment | | Control | |  | Treatment | | Control | |
|  |  |  | N | n | N | n |  | N | n | N | n |
| Barer 1988 atenolol | 18 | 11 | 18 | 4 | 11 | 4 |  | 16 | 8 | 10 | 5 |
| Barer 1988 propanolol | 16 | 11 | 16 | 7 | 10 | 3 |  | 16 | 11 | 10 | 5 |
| Barer 1988/50 mg | 102 | 50 | 102 | 37 | 50 | 12 |  | 101 | 45 | 50 | 21 |
| Barer 1988/80 mg | 101 | 50 | 100 | 33 | 50 | 12 |  | 100 | 48 | 50 | 22 |
| ASCLEPIOS 1990 | 120 | 114 | 116 | 21 | 114 | 19 |  | 116 | 47 | 114 | 44 |
| Limburg 1990 | 12 | 14 | 12 | 3 | 14 | 5 |  | 12 | 3 | 14 | 7 |
| Norris 1994 | 96 | 93 | 96 | 29 | 93 | 33 |  | 90 | 39 | 79 | 42 |
| Bogousslavsky 1990 | 24 | 28 | 24 | 0 | 28 | 1 |  | 24 | 6 | 28 | 4 |
| Kaste 1994/120 mg | 176 | 174 | 176 | 29 | 174 | 22 |  | 175 | 44 | 172 | 31 |
| Lowe 1993 | 56 | 56 | 56 | 15 | 56 | 12 |  | 40 | 25 | 46 | 18 |
| Paci 1989/120 mg | 19 | 22 | 19 | 0 | 22 | 0 |  | 19 | 1 | 22 | 4 |
| Squire 1996 | 75 | 72 | 75 | 12 | 72 | 17 |  | 69 | 32 | 63 | 32 |
| VENUS 1995 | 225 | 229 | 225 | 30 | 229 | 32 |  | 223 | 63 | 225 | 57 |
| Lees 1995 | 30 | 30 | 30 | 6 | 30 | 7 |  | 30 | 9 | 30 | 12 |
| IMAGES Pilot | 26 | 25 | 26 | 3 | 25 | 6 |  | 26 | 12 | 25 | 11 |
| Muir 1995 | 19 | 6 | 19 | 1 | 6 | 0 |  | 19 | 3 | 6 | 1 |
| Strand 1984 | 13 | 13 | 13 | 2 | 13 | 3 |  | 13 | 6 | 13 | 9 |
| PRISTINE | 313 | 307 | 307 | 49 | 303 | 41 |  | 307 | 184 | 303 | 182 |
| Steiner 1986 | 55 | 45 | 55 | 21 | 45 | 16 |  | 55 | 38 | 45 | 33 |
| Herrschaft 1988 | 24 | 20 | 23 | 0 | 17 | 0 |  | 23 | 8 | 17 | 10 |
| Huczynski 1988 | 15 | 15 | 15 | 4 | 15 | 1 |  | 14 | 4 | 15 | 2 |

**Supplementary Table C3** Effect size and approximate within-study covariance matrices for bivariate meta-analyses of acute stroke data [4]

|  | Bivariate meta-analysis of SBP and DBP | | | | |  | Bivariate meta-analysis of death (D) and ‘death or disability’ (DD) | | | | |
| --- | --- | --- | --- | --- | --- | --- | --- | --- | --- | --- | --- |
| Study | Mean difference in SBP | Mean difference in DBP | Var(MD_SBP_) | Var(MD_DBP_) | Cov(MD_SBP_, MD_DBP_) |  | Log OR for D | Log OR for DD | Var(logOR_D_) | Var(logOR_DD_) | Cov(logOR_D_, logOR_DD_) |
| Barer 1988 atenolol | -2.47 | -3.44 | 87.99 | 27.85 | 34.81 |  | -0.693 | 0 | 0.714 | 0.650 | 0.504 |
| Barer 1988 propanolol | 1.61 | -0.34 | 128.50 | 42.48 | 52.31 |  | 0.596 | 0.788 | 0.730 | 0.691 | 0.447 |
| Barer 1988/50 mg | -8.16 | -6.44 | 17.50 | 6.89 | 7.75 |  | 0.589 | 0.104 | 0.152 | 0.122 | 0.098 |
| Barer 1988/80 mg | -3.17 | -3.41 | 16.49 | 6.83 | 7.52 |  | 0.444 | 0.161 | 0.155 | 0.121 | 0.091 |
| ASCLEPIOS 1990 | -0.15 | -2.39 | 11.20 | 3.187 | 4.23 |  | 0.100 | 0.080 | 0.121 | 0.073 | 0.053 |
| Limburg 1990 | -9.83 | 1.93 | 171.72 | 65.03 | 74.53 |  | -0.511 | -1.099 | 0.756 | 0.730 | 0.667 |
| Norris 1994 | -16.25 | -11.88 | 11.60 | 3.34 | 4.39 |  | -0.24 | -0.395 | 0.096 | 0.096 | 0.079 |
| Bogousslavsky 1990 | -7 | 1 | 33.14 | 9.36 | 12.27 |  | -0.554 | 0.693 | 3.09- | 0.514 | 0.426 |
| Kaste 1994/120 mg | -1.9 | -4.9 | 8.25 | 2.64 | 3.30 |  | 0.310 | 0.424 | 0.093 | 0.070 | 0.064 |
| Lowe 1993 | 4.17 | 3.81 | 20.54 | 5.65 | 7.64 |  | 0.294 | 0.953 | 0.197 | 0.198 | 0.14 |
| Paci 1989/120 mg | -9 | -2.4 | 33.58 | 9.63 | 12.73 |  | 0.150 | -1.386 | 4.101 | 1.361 | 1.25 |
| Squire 1996 | -2.13 | -1.18 | 16.20 | 4.66 | 6.17 |  | -0.484 | -0.177 | 0.176 | 0.122 | 0.082 |
| VENUS 1995 | 0.53 | -1.65 | 6.28 | 1.62 | 2.27 |  | -0.054 | 0.149 | 0.075 | 0.046 | 0.039 |
| Lees 1995 | 7.96 | 2.71 | 52.30 | 20.98 | 23.50 |  | -0.197 | -0.442 | 0.395 | 0.298 | 0.248 |
| IMAGES Pilot | -8.59 | -9.18 | 41.67 | 8.69 | 13.30 |  | -0.884 | 0.087 | 0.596 | 0.317 | 0.214 |
| Muir 1995 | -8.32 | -7.55 | 148.77 | 73.07 | 73.96 |  | -0.492 | -0.065 | 3.237 | 1.596 | 1.352 |
| Strand 1984 | 18.04 | 3.79 | 126.42 | 28.03 | 42.06 |  | -0.501 | -0.965 | 1.024 | 0.671 | 0.341 |
| PRISTINE | 1.37 | 1.84 | 3.96 | 1.03 | 1.43 |  | 0.194 | -0.005 | 0.053 | 0.027 | 0.013 |
| Steiner 1986 | -7.41 | -4.16 | 27.67 | 8.99 | 11.17 |  | 0.113 | -0.207 | 0.174 | 0.199 | 0.09 |
| Herrschaft 1988 | 2.4 | -0.7 | 40.71 | 12.81 | 16.11 |  | -0.310 | -0.985 | 4.105 | 0.435 | 0.225 |
| Huczynski 1988 | -5.93 | 0.53 | 100.06 | 28.32 | 36.82 |  | 1.627 | 0.956 | 1.412 | 0.927 | 0.886 |

SBP = systolic blood pressure, DBP = diastolic blood pressure, D = death, DD = death or disability, MD = mean difference OR = odds ratio

1. To whom correspondence should be addressed. y.wei@ctu.mrc.ac.uk [↑](#footnote-ref-2)
